# Synthesis and Biological Evaluation of New Hydrazone Derivatives of Quinoline and Their Cu(II) and Zn(II) Complexes against *Mycobacterium tuberculosis*

**DOI:** 10.1155/2015/153015

**Published:** 2015-11-25

**Authors:** Mustapha C. Mandewale, Bapu Thorat, Dnyaneshwar Shelke, Ramesh Yamgar

**Affiliations:** ^1^P.G. and Research Centre, Department of Chemistry, Government of Maharashtra, Ismail Yusuf College of Arts, Science and Commerce, Jogeshwari (East), Mumbai 400 060, India; ^2^Department of Chemistry, Chikitsak Samuha's Patkar-Varde College of Arts, Science and Commerce, Goregaon (West), Mumbai 400 062, India

## Abstract

A new series of quinoline hydrazone derivatives and their metal complexes have been synthesized and their biological properties have been evaluated against *Mycobacterium tuberculosis* (H37 RV strain). Most of the newly synthesized compounds displayed 100% inhibitory activity at a concentration of 6.25–25 *μ*g/mL, against *Mycobacterium tuberculosis*. Fluorescence properties of all the synthesized compounds have been studied.

## 1. Introduction

The tuberculosis also known as TB and “white plaque” is caused by infection with different species of bacteria including* Mycobacterium tuberculosis*,* Mycobacterium africanum*,* Mycobacterium bovis*,* Mycobacterium caprae*,* Mycobacterium canettii*,* Mycobacterium pinnipedii*, and* Mycobacterium microti*. Other factors that have also contributed include population expansion, poor case detection and slow cure rates in developing countries, active transmission in overcrowded hospitals, drug abuse, and homelessness. Successful treatment of fully susceptible TB depends on combination of drugs and duration of therapy, cost, and drug's side effects. Treatment failure is due to multiple factors including incomplete or short duration therapy and results in persistence of the disease mainly due to emergence of drug resistance. Primary resistance occurs when the resulting* Mycobacterium tuberculosis* strain is transmitted to a new host and it causes tuberculosis that is already resistant to the drugs used for treatment of previous host [1, 2].

Major factors associated with drug resistance development include nonadherence to therapy due to multiple factors such as high cost of drugs, long duration of therapy and combination of multiple drugs used in treatment regimens and drug related coinfection with HIV, adverse reactions, prior history of treatment, and treatment failures with antituberculosis (anti-TB) drugs [3–6]. Simultaneous treatment of HIV-tuberculosis coinfection may lead to malabsorption and suboptimal therapeutic blood levels of Rifampin (RMP) and Isoniazid (INH) that facilitate the development of drug resistant TB and multiple drug resistant tuberculosis (MDR-TB). The malabsorption of antituberculosis drugs may also occur in patients having some other diseases in association [7, 8].

The treatment of tuberculosis involves first-line drugs including Streptomycin, Isoniazid (INH), Rifampin (RMP), Ethambutol, and Pyrazinamide. The second-line treatments of tuberculosis involve the application of p-aminosalicylic acid, Ethionamide, Cycloserine, Azithromycin, Clarithromycin, and Fluoroquinolones. However, the main complication associated with TB therapy is the poor assent with the long duration of the treatment and mainly with the drugs used to treat MDR-TB that are increasing resistance, expensive, relatively ineffective, and having long duration of treatment. The Isoniazid is one of the commonly used and effective antitubercular drugs, but recently because of the appearance of MTB resistant strains many attempts have been made to explain the mechanism of interaction of this drug and the origin of drug resistance and research its novel/new drugs for the treatment of tuberculosis. Therefore it is still a challenge for the researchers to develop drugs of more efficiency, more effective with less toxicity to treat signs and symptoms of tuberculosis.

On the side the various pharmacological properties of quinoline and their derivatives attracted great attention in the last few decades because of their vast occurrence in natural products and drugs [9]. A large number of efforts have been made to develop quinoline-based compounds as important antitubercular agent, which is active on different clinically approved therapeutic targets showing excellent therapeutic potency in the past decade. The literature survey shows that 5 to 6 membered heterocyclic compounds containing a quinoline ring in a linear fashion were found to exhibit strong anticancer and antimicrobial activities [10, 11]. Various derivatives of quinoline have been employed in the synthesis of antifungal, antihypertensive, and antibacterial drugs. Recently interest in the study of Schiff base hydrazones has been increasing because of their antitumour, antitubercular, and antimicrobial activities. Schiff base hydrazones play an important role in inorganic chemistry as they easily form stable coordination complexes with most transition series metal ions. The developments in the field of bioinorganic chemistry have increased the interest in hydrazone complexes, because it has been known that many of these complexes may act as lead for biologically important species [12–16].

## 2. Experimental

The chemicals and solvents used in this work were of analytical grade and purchased from Merck and Sigma-Aldrich Chemicals. The zinc chloride, copper chloride, and DMF (N,N-dimethylformamide) were purchased from SD Fine Chemicals.

### 2.1. Synthesis of Schiff Base Hydrazones from 6-Fluoro-2-hydroxyquinoline-3-carbaldehyde

6-Fluoro-2-hydroxyquinoline-3-carbaldehyde was synthesized by Vilsmeier-Haack reaction starting with 4-fluoroacetanilide as per reported method [17, 18].

6-Fluoro-2-hydroxyquinoline-3-carbaldehyde (0.200 g, 0.0015 moles) was dissolved in 5 mL ethanol and compounds 1a–1e (0.0015 moles) were added. A drop of glacial acetic acid was added as a catalyst for the reaction. The reaction mixture was refluxed for half an hour. The reaction mixture was cooled in ice bath and precipitated product was filtered. The product was then dried in oven. Structures of synthesized hydrazone derivatives have been shown in Figure 10 and Table 1.

#### 2.1.1. Preparation of N′-[(E)-(6-Fluoro-2-hydroxyquinoline-3-yl)methylidene]pyridine-3-carbohydrazide [2a]

M.P.: 293–295°C; UV *λ*_max_: 383 nm; MS [M + H]: 311.59; FTIR (KBr cm^−1^): 3208 (phenolic -OH), 3073 (-N-H amide), 1660 (azomethine -CH=N-), 1625 (C=O amide), 1425 (phenolic C-O), 1294 (C-F quinoline); ^1^H NMR (300 MHz, DMSO-d_6_) *δ*: 7.35–7.42 (m, 2H), 7.55 (s, 1H), 7.75–7.78 (d, 1H), 8.25–8.28 (d, 1H), 8.49 (s, 1H), 8.69–8.75 (m, 2H), 9.08 (s, 1H), 12.09 (s, 1H), 12.16 (s, 1H); ^13^C NMR (75 MHz, DMSO-d_6_) *δ*: 163.01 (-C=O amide), 161.63 (-C-O phenolic), 150.02 (-C-F quinoline), 148.75, 148.24, 147.26, 146.23 (-C=N- azomethine), 133.28, 130.99, 130.66, 130.50, 127.90, 126.63, 124.99, 119.43, 110.5; elemental analysis: observed (calculated): C 61.97% (61.93%), H 3.66% (3.57%), N 18.20% (18.06%).

#### 2.1.2. Preparation of N′-[(E)-(6-Fluoro-2-hydroxyquinoline-3-yl)methylidene]pyridine-4-carbohydrazide [2b]

M.P.: >300°C; UV *λ*_max_: 388 nm; MS [M − H]: 309.27; FTIR (KBr cm^−1^): 3488 (phenolic -OH), 3153 (N-H amide), 3025 (aromatic C-H), 1650 (imine -CH=N-), 1630 (C=O amide), 1427 (phenolic C-O), 1288 (C-F quinoline); ^1^H NMR (300 MHz, DMSO-d_6_) *δ*: 7.32–7.45 (m, 2H), 7.77–7.85 (m, 3H), 8.50 (s, 1H), 8.71 (m, 2H), 8.77 (s, 1H), 12.13 (s, 1H), 12.26 (s, 1H); ^13^C NMR (75 MHz, DMSO-d_6_) *δ*: 164.8 (-C=O amide), 162.52 (-C-O phenolic), 152.32 (C-F quinoline), 150.01, 147.26, 140.97 (-C=N- azomethine), 134.57, 131.02, 131.52, 127.43, 125.71, 119.46, 118.21, 110.5; elemental analysis: observed (calculated): C 61.98% (61.93%), H 3.72% (3.57%), N 18.17% (18.06%).

#### 2.1.3. Preparation of N′-[(E)-(6-Fluoro-2-hydroxyquinoline-3-yl)methylidene]-6-methylpyridine-3-carbohydrazide [2c]

M.P.: >300°C; UV *λ*_max_: 385 nm; MS [M + H]: 325.16; FTIR (KBr cm^−1^): 3444 (phenolic -OH), 3228 (N-H amide), 2933 (aromatic C-H), 2886 (aliphatic C-H), 1662 (imine -CH=N-), 1628 (C=O amide), 1438 (phenolic C-O), 1234 (C-F quinoline); ^1^H NMR (300 MHz, DMSO-d_6_) *δ*: 2.54 (s, 3H), 7.36–7.43 (m, 3H), 7.76–7.79 (m, 1H), 8.17–8.19 (m, 1H), 8.49 (s, 1H), 8.70 (s, 1H), 8.97 (s, 1H), 12.16 (s, 2H); ^13^C NMR (75 MHz, DMSO-d_6_) *δ*: 163.31 (-C=O amide), 160.40 (-C-O- phenolic), 155.47 (-C-F quinoline), 158.48, 147.94, 145.66, 141.29 (-C=N- azomethine), 131.29, 131.06, 130.01, 128.69, 126.72, 125.23, 122.08, 117.93, 112.4, 23.97 (-CH_3_ pyridine); elemental analysis: observed (calculated): C 62.87% (62.96%), H 4.13% (4.04%), N 17.34% (17.28%).

#### 2.1.4. Preparation of 2-[(7-Bromo-2,3-dihydro-1H-inden-4-yl)oxy]-N′-[(E)-(6-fluoro-2-hydroxyquinoline-3-yl)methylidene]acetohydrazide [2d]

M.P.: >300°C; UV *λ*_max_: 382 nm; MS [M + H]: 458.00; FTIR (KBr cm^−1^): 3538 (phenolic -OH), 3432 (N-H amide), 3002 (aromatic C-H), 2848 (aliphatic C-H), 1656 (imine -CH=N-), 1627 (C=O amide), 1425 (phenolic C-O), 1263 (C-F quinoline); ^1^H NMR (300 MHz, DMSO-d_6_) *δ*: 2.85–2.97 (m, 6H), 4.48 (s, 2H), 7.24–7.47 (m, 2H), 7.59–7.62 (m, 1H), 7.74–7.81 (m, 1H), 8.23 (s, 1H), 8.42 (s, 1H), 8.51 (s, 1H), 11.82 (s, 1H), 12.13 (s, 1H); ^13^C NMR (75 MHz, DMSO-d_6_) *δ*: 168.50 (-C=O amide), 162.75 (C-O- phenolic), 159.14 (C-F quinoline), 155.80, 154.55, 146.26, 143.72 (-C=N- azomethine), 135.14, 133.59, 131.79, 130.06, 127.43, 125.93, 122.20 (-C-Br), 120.93, 111.97, 109.95, 67.83 (-CH_2_-O-), 29.83 (CH_2_ aliphatic), 29.77 (CH_2_ aliphatic), 25.78 (CH_2_ aliphatic); elemental analysis: observed (calculated): C 55.50% (55.04%), H 3.69% (3.74%), N 9.20% (9.17%).

#### 2.1.5. Preparation of 2-(2,3-Dihydro-1H-inden-4-yloxy)-N′-[(E)-(6-fluoro-2-hydroxyquinoline-3-yl)methylidene]acetohydrazide [2e]

M.P.: >300°C; UV *λ*_max_: 381 nm; MS [M + H]: 380.44; FTIR (KBr cm^−1^): 3193 (phenolic -OH), 3064 (N-H amide), 2950 (aromatic C-H), 2854 (aliphatic C-H), 1668 (imine -CH=N-), 1625 (C=O amide), 1428 (phenolic C-O), 1232 (C-F quinoline); ^1^H NMR (300 MHz, DMSO-d_6_) *δ*: 1.99 (m, 2H), 2.83 (m, 4H), 4.65 (s, 2H), 6.60–6.65 (m, 1H), 6.82 (m, 1H), 7.05 (m, 1H), 7.33–7.42 (m, 2H), 7.60–7.62 (m, 1H), 8.22 (s, 1H), 8.42 (s, 1H), 11.76 (s, 1H), 12.10 (s, 1H); ^13^C NMR (75 MHz, DMSO-d_6_) *δ*: 168.40 (-C=O amide), 162.96 (-C-O- phenolic), 159.81 (-C-F quinoline), 154.90, 147.35, 146.95 (-C=N- azomethine), 137.5, 135.14, 130.99, 130.8, 130.66, 127.90, 126.63, 125.01, 119.43, 113.72, 110.5, 66.86, 32.61 (CH_2_ aliphatic), 29.67 (CH_2_ aliphatic), 25.38 (CH_2_ aliphatic); elemental analysis: observed (calculated): C 66.52% (66.48%), H 4.70% (4.78%), N 11.21% (11.08%).

### 2.2. Synthesis of Cu(II) and Zn(II) Complexes from 2a–2e

The solution of metal salt [ZnCl_2_, CuCl_2_] dissolved in ethanol was added gradually to a stirred ethanolic solution of the Schiff base hydrazones [2a–2e], in the molar ratio 1 : 2. The reaction mixture was further stirred for 2–4 hr at 60°C. Then it was cooled in ice bath to ensure the complete precipitation of the formed complexes. The precipitated solid complexes were filtered and washed four times with water. Finally, the complexes were washed with diethyl ether and dried in vacuum desiccators over anhydrous CaCl_2_. Structures of synthesized complexes have been shown in Figure 11 and Table 2.

## 3. Results and Discussion

All the synthesized hydrazone ligands 2a–2e and their Cu^2+^ and Zn^2+^ complexes 3a–3j are stable at room temperature and are nonhygroscopic in nature. The metal complexes are insoluble in H_2_O but are soluble in DMF. The spectral characterizations of synthesized compounds confirm the suggested structures of the hydrazones as well as their metal complexes. The elemental analysis, physical properties, and spectral data of the ligand and complexes are summarized below.

### 3.1. ^1^H NMR Spectra

^1^H NMR spectra were recorded on Varian-NMR-Mercury 300 MHz instrument. The DMSO-d_6_ was used as a solvent with TMS (tetramethylsilane) as an internal standard. The chemical shifts are expressed as *δ* values (ppm). The ^1^H NMR spectra of the hydrazones 2a–2e were recorded in DMSO-d6 solvent over the range of 0–16 ppm and shown in Table 3. The disappearance of the singlet at 10.23 ppm and appearance of new singlet peak in the range of 8.59–9.08 ppm are assignable to the azomethine protons which confirms the condensation of hydrazones 1a–1e and 6-fluoro-2-hydroxyquinoline. Also a set of multiplets observed in the range 6.60–8.40 ppm is ascribed to the aromatic protons in all the compounds. The peak observed in the range of 11.76–12.13 ppm in the hydrazones is attributable to the -OH of quinoline at 2nd position. A sharp singlet at 12.10–12.26 ppm observed in the hydrazones is due to -NH of amide carbonyl.

### 3.2. ^13^C NMR Spectra

In the ^13^C spectra azomethine carbon atom appeared most downfield as reported in literature values; in the hydrazones (2a–2e) it was observed in the range of 140.97–146.95 ppm shown in Table 4. The phenolic carbon appears in the range of 160.40–162.75 ppm. The amidic carbon from hydrazide gives signal in the range of 163.01–168.50 ppm. The ^13^C spectral analysis confirms the formation of the hydrazone derivatives 2a–2e.

### 3.3. Mass Spectra

The formation of imine is confirmed by the presence of intense molecular ion peak in the mass spectra of hydrazone derivatives (2a–2e). Spectral evaluation predicts the molecular weights of the desired hydrazone compounds.

### 3.4. IR Spectra

The infrared spectra were recorded on FTIR-7600 Lambda Scientific Pty. Ltd. using KBr pellets. From the interpretation of IR spectra we get valuable information regarding the nature of functional group present in the hydrazone derivatives (2a–2e) and metal complexes (3a–3j). In the IR spectra of hydrazones the imine group (-HC=N-) and hydroxyl group show strong peak in the regions of 1625–1630 cm^−1^ and 3193–3538 cm^−1^, respectively. All metal complexes show broad peak in the region of 3200–3400 cm^−1^ due to coordinated water molecules.

In order to study the bonding mode of hydrazone ligand to the central metal atom the IR spectra of the free hydrazones were compared with the spectra of the complexes. The important IR bands and their assignments are listed in Tables 5 and 6.

The phenolic -OH band appears at 3193–3588 cm^−1^ which disappears in IR spectra of the metal complexes; however new broad peak has been observed at 3200–3400 cm^−1^ due to coordinated water molecules which confirms the complexation of hydrazones with central metal atom through phenolic -OH. The IR spectra of all the metal complexes show prominent band at about 501–599 cm^−1^ due to *υ*_M-N_ stretching. The low frequency region of the spectra indicated the presence of two new medium intensity bands at about 449–482 cm^−1^ due to *υ*_M-O_ vibrations and at 501–599 cm^−1^ due to *υ*_M-N_ vibrations.

### 3.5. Molar Conductivity Measurements

From the mathematical relation Λ*m* = *K*/*C* the molar conductance of the metal complexes (Λ*m*) can be calculated by dissolving in proper solvent where *C* is the molar concentration of the metal complex solutions [19]. The metal complexes (3a–3j) were dissolved in DMF to prepare 10^−3^ M of their solutions. The molar conductivities were measured at 25 ± 2°C. The study shows negligible molar conductance values for metal complexes 3a–3j (2.90–7.30 Ω^−1^ mol^−1^ cm^2^), indicating that the complexes are nonelectrolytes. The results are represented in Table 7.

### 3.6. Elemental Analysis of Metal Complexes

**Figure 1:**
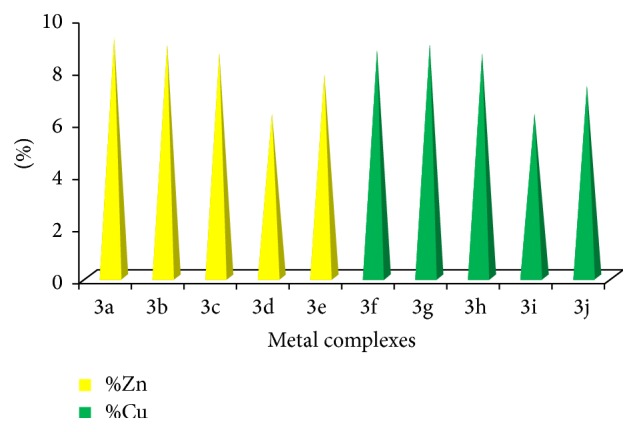
Showing comparative % of Zn and Cu contents in metal complexes 3a–3j.

The quantitative estimation of Cu(II) and Zn(II) has been done by complexometric titration with standard EDTA solution. In a titration an accurately known mass of metal complex is dissolved in an aqueous solution by chemical treatment such as acid-digestion of solid metal complex samples and diluted with high purity water to an accurately known volume. Then an accurately known volume of the aliquot is pipetted into a titration vessel and the analyte of interest is carefully titrated with a standardized EDTA solution to the endpoint of the titration [20]. The observed results are represented in Table 8 and Figure 1.

From the experimental study it is clear that practical observations are in good agreement with the theoretical values calculated for 1 : 2 ratio of metal : ligand stoichiometry. Regarding the above explanation of the results of various spectroscopic details, it may be concluded that the proposed geometry for the transition metal complexes with general formula ML_2_·2H_2_O is octahedral for Zn^+2^ and Cu^+2^ complexes. The probable structures are shown in Table 2.

### 3.7. Structure Activity Relationship Study (SAR)

The Cresset software Forge is a molecular design and SAR (structure activity relationship) interpretation tool that generates and uses molecular alignments as a way to make meaningful comparisons across chemical series. The interaction between a ligand and a protein involves electrostatic fields and surface properties (e.g., hydrogen bonding and hydrophobic surfaces). Two molecules which both bind to a common active site tend to make similar interactions with the protein and hence have highly similar field properties.

Accordingly, using these properties to describe molecules is a powerful tool for the medicinal chemist as it concentrates on the aspects of the molecules that are important for biological activity. In Forge, molecules can be aligned by using the fields of the molecules, by using shape properties, or by using a common substructure. Using the fields gives a “protein's view” of how the molecules would line up in the active site, generating ideas on how molecules with different structures could interact with the same protein. Using substructure or common shape properties shows how the fields around a single chemical series vary with activity and in many cases these can be automatically examined to give a 3D structure active relationship (SAR) with predictive power for new ideas for synthesis.

#### 3.7.1. Interpretation of Field Point Patterns

Molecules bear different types of field points. Larger field points represent stronger points of potential interaction. Throughout Cresset's software the blue points are negative field points which like to interact with positives/H-bond donors on a protein, whereas red points are positive field points which like to interact with negatives/H-bond acceptors on a protein. Similarly the yellow points are van der Waals surface field points which describe possible surface/van der Waals interactions. It can be seen that ionic groups give rise to the strongest electrostatic fields. Hydrogen bonding groups also give strong electrostatic fields. Aromatic groups encode both electrostatic and hydrophobic fields. Aliphatic groups such as the methyl or cyclopentyl group give rise to hydrophobic and surface points but are essentially electrostatically neutral.

To generate these fields, we use XED (Extended Electron Distribution) molecular mechanics force field, which uses off-atom sites to more accurately describe the electron distribution in a molecule, as opposed to other force fields where charges are placed at the atomic nuclei only.

**Figure 2:**
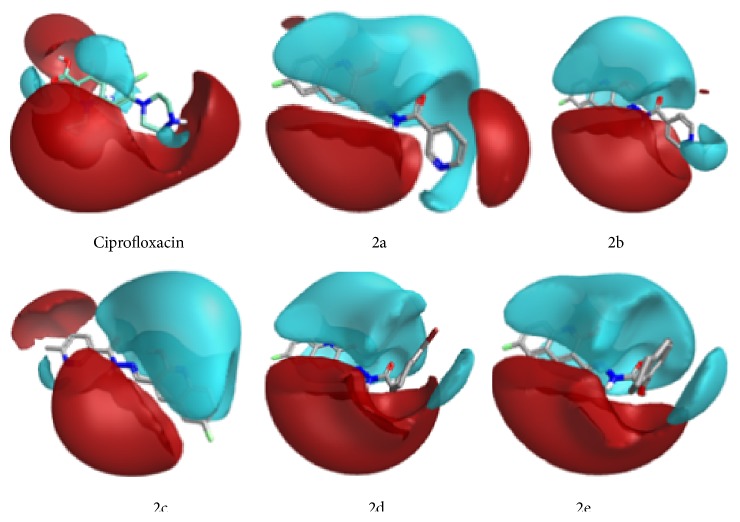
Showing different electrostatic regions of products 2a–2e.

**Figure 3 fig3:**
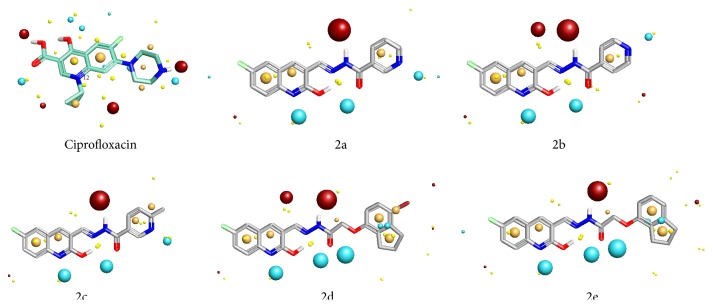
Showing point charges of products 2a–2e.

**Figure 4:**
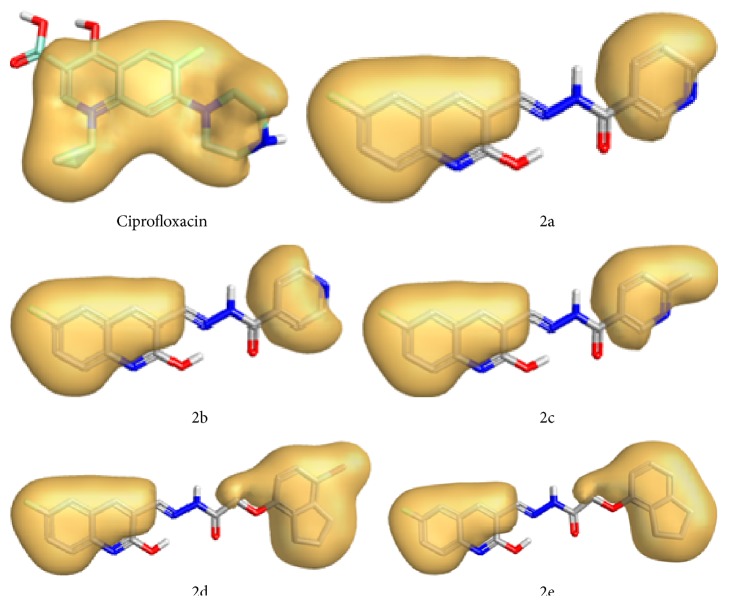
Hydrophobic regions for products 2a–2e.

For SAR study we have selected ciprofloxacin as reference compound as it shows strong antitubercular activity against* Mycobacterium tuberculosis*. The bactericidal action of ciprofloxacin results from inhibition of the enzymes* topoisomerase II* (DNA-*gyrase*) and* topoisomerase IV*, which are required for bacterial DNA replication, transcription, repair, strand supercoiling repair, and recombination. By keeping in mind two molecules which both bind to a common active site of receptor and tend to make similar interactions with the protein and hence have highly similar field properties, we have compared the field properties of the ciprofloxacin with synthesized hydrazone derivatives 2a–2e. This comparison strengthens the correlation between the theoretical and observed activities of the target compounds. We have calculated structure activity score of compounds 2a–2e compared with ciprofloxacin. The structure activity score for 2a is 0.556, for 2b is 0.536, for 2c is 0.536, for 2d is 0.505, and for 2e is 0.526. All hydrazones have comparable positive field regions, but their negative field regions are largely different from that of ciprofloxacin (Figures 2 [Fig fig3]–4). This difference in the field regions affects the activity. Compounds 2b and 2e show least negative field regions and are close to that of ciprofloxacin and hence show greater activity against* Mycobacterium tuberculosis*.

### 3.8. Biological Assay

#### 3.8.1. Antitubercular Studies

The antitubercular activity of the hydrazone ligands and their metal complexes was tested against* Mycobacterium tuberculosis* (H37 RV strain) ATCC number 27294 to find out their potency as antimicrobial agent by MIC method (minimum inhibitory concentration).

#### 3.8.2. Microbiological Method

The antibacterial activity of hydrazones and their metal complexes was tested against* Mycobacterium tuberculosis* using Microplate Alamar Blue Assay (MABA) (Figure 1) [21]. This methodology is nontoxic and reagents used are thermally stable. It also shows good correlation with proportional and BACTEC radiometric method and reproducible results.

To minimize the evaporation of medium in the test wells during incubation, 200 *μ*L of sterile deionized water was added to all outer perimeter wells of sterile 96-well plate. The 96-well plate received 100 *μ*L of the Middlebrook 7H9 broth and serial dilution of compounds was made directly on plate. The concentration of the test sample was prepared between 100 and 0.8 *μ*g/mL ranges. These plates were covered and sealed with paraffin and incubated at 37°C for five days. Then in the next step, 25 *μ*L of freshly prepared 1 : 1 mixture of Alamar Blue Reagent Tween 10% and Tween 80% was added to the plate and incubated for 24 hrs in incubator. A blue color in the well indicates bacterial growth whereas pink color shows growth of bacteria. From this experiment the MIC can be defined as lowest drug concentration which prevented the color change from blue to pink (Figure 1).

#### 3.8.3. *In Vitro* Antimicrobial Activity

When we compare MIC values of hydrazone and its complexes, it indicates that metal complexes exhibit higher antimicrobial activity than the free hydrazone ligands and the same is represented from the results given in Table 9.

**Figure 5:**
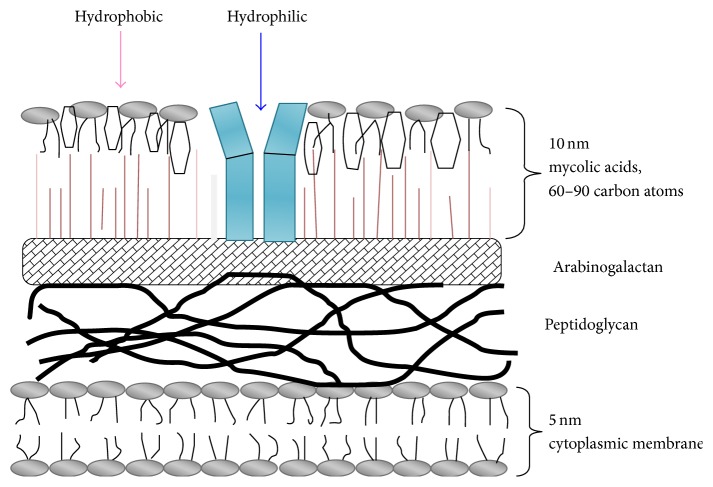
Schematic diagram of mycobacterium cell wall.

There was moderate to good antibacterial activity observed against* Mycobacterium tuberculosis* (H37 RV strain). It was in the range of MIC values of 6.25–25.00 *μ*g/mL concentration compared to standard antibiotic ciprofloxacin having MIC of 3.12 *μ*g/mL, Pyrazinamide having MIC of 3.12 *μ*g/mL, and Streptomycin having MIC of 6.25 *μ*g/mL. These observations may be because of presence of active pharmacophore present in the molecular structure of the hydrazone and metal complexes like fluorine substituted heterocyclic ring of quinoline moiety, pyridine heterocyclic ring, imine double bond between carbon and nitrogen, and well positioned hydroxyl group. These structural scaffolds might interfere in the mechanism of cell multiplication and hence stop further growth of* Mycobacterium tuberculosis*. All the studied samples are showing different potency due to the effective barrier of an outer cell wall membrane of* Mycobacterium tuberculosis* for entry of external substances like test compounds under this study. The schematic diagram of mycobacterial cell wall is represented in Figure 5.

Metal complexes showed greater activity as compared with hydrazone ligand. This may be probably because of the greater lipophilic character of the complexes. This increased activity of the metal complexes can be explained on the basis of coordination theory [22]. According to Overtone's concept of cell permeability, only lipid soluble materials favour the passage of the lipid membrane that surrounds the cell. Therefore the extent of lipid solubility of a molecule is an important factor which controls the antimicrobial activity. According to the molecular theory of coordination of metal orbital overlap with ligand orbitals, this reduces the positive charge on the metal ion by accepting the electrons from donor groups of the hydrazone ligand [23, 24]. Thus the donation of the electrons from ligand to metal also favours the increased delocalization of the *π*-electrons through entire coordinating rings. This results in increased lipophilicity of the metal complexes. Therefore the enhanced lipophilicity facilitates the passage of the complexes across the lipid cell membrane of the bacteria and hence it can block the metal binding sites on different enzymes of bacteria [25]. Also these metal complexes disturb the respiration process of the cell and thereby prevent the synthesis of proteins. If the synthesis of proteins is blocked, then formation of bacterial cell wall is not possible and hence finally it results in cell death and therefore restricts further growth of the bacteria [26]. According to one more possible mechanism, these complexes might be interacting with the DNA gyrase enzyme which is essential for DNA multiplication step. The DNA gyrase is inhibited by metal complexes which alter the multiplication of bacterial cells, ultimately resulting in death of the bacteria [27, 28].

However, in case of test samples 3f and 3h complexes showed good activity up to MIC value of 6.5 *μ*g/mL and 3c, 3e, 3g, and 3i complexes showed activity up to MIC value of 12.5 *μ*g/mL. This could be due to coordination of central metal atom to form a specific complex with cell wall protein and ultimately interfering in cell wall synthesis of* Mycobacterium tuberculosis* during cell mitosis phase of multiplication. The observed results of the test compounds indicate the future potential for the development of metal coordination complexes to solve the limitations due to currently available antituberculosis agents to treat multiple drug resistant tuberculosis.

### 3.9. Fluorescence Study

**Figure 6:**
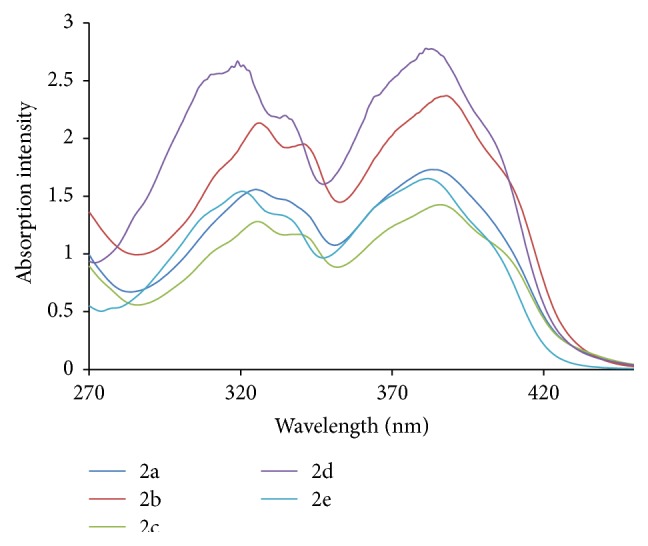
Electronic spectra of hydrazones (2a–2e).

**Figure 7 fig7:**
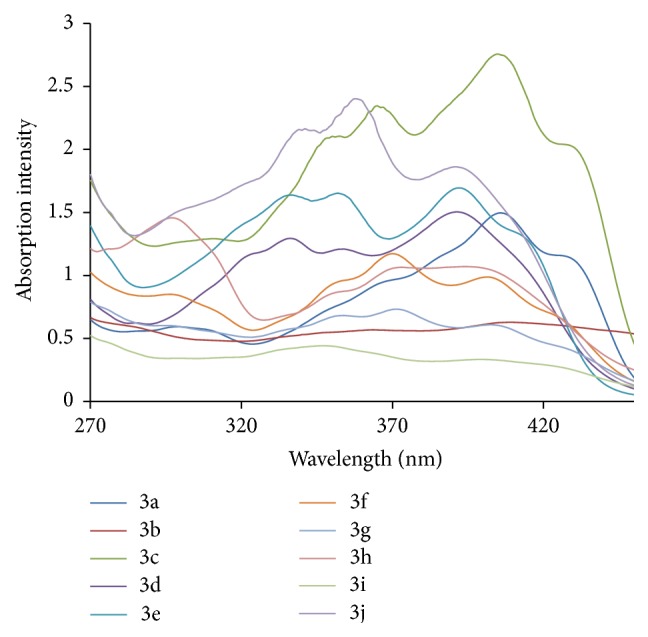
Electronic spectra of metal complexes (3a–3j).

**Figure 8 fig8:**
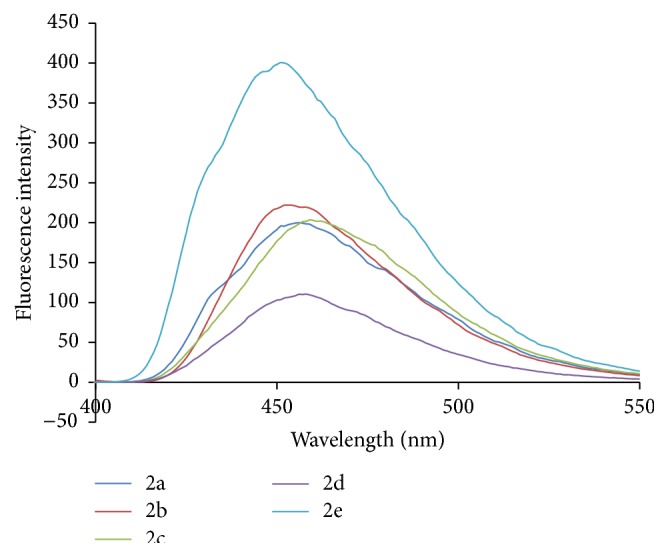
Fluorescence spectra of hydrazones (2a–2e).

**Figure 9:**
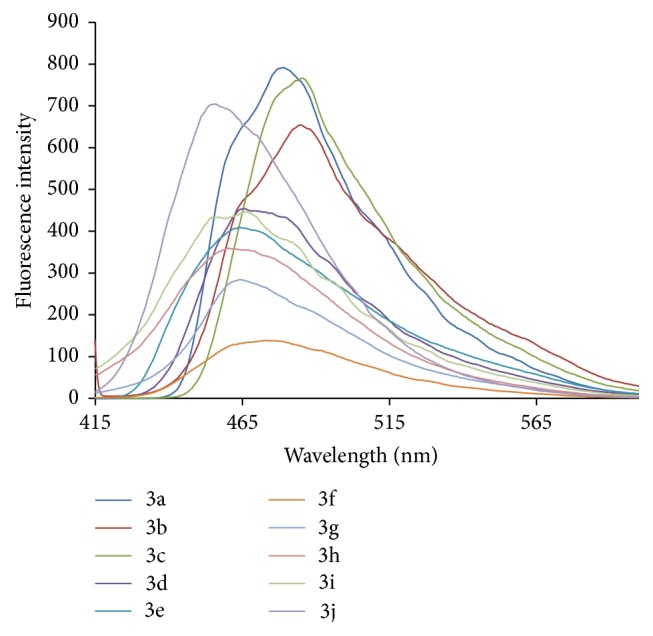
Fluorescence spectra of metal complexes (3a–3j).

**Figure 10:**
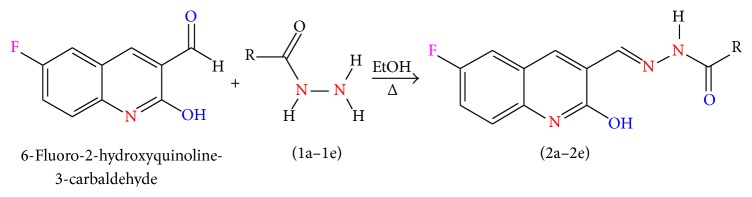


**Figure 11:**
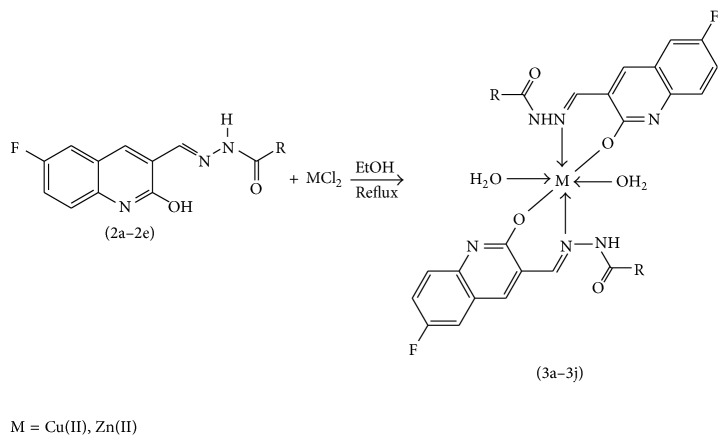


The UV-Visible absorption spectra were recorded with UV spectrophotometer model Shimadzu UV-1800. The path length of the measurements was 1 cm. The fluorescence study was done on a Spectrofluorophotometer model Shimadzu RF-5301pc having 1 cm path length. The concentration 200 ppm of ligand and metal complexes which was in DMF (N,N-dimethylformamide) was prepared for study. Figures 6 [Fig fig7] [Fig fig8]–9 show absorption as well as emission spectra. The instrumental results are summarized in Table 10.

All the ligands in UV-Visible spectra exhibit bands around 381–388 nm. The broad intense band around 280 nm in the ligands can be assigned to intraligand n-p^*∗*^ transition associated with the azomethine linkage. This band experiences red shift in all the complexes. The bands at around 390–408 nm are attributed to the ligand to metal charge transfer transitions.

The emission spectra of compounds 2a–2e showed emission band in the range of 451–459 nm and compounds 3a–3j showed emission band in the range of 456–485 nm. Compounds 3a, 3b, 3c, and 3j showed higher emission intensity. Complex formation of hydrazones induces marked hyperchromic and bathochromic shifts. The Zn(II) complexes showed intense fluorescent properties as compared to Cu(II) complexes and their parent ligands.

## 4. Conclusions

To assess the antimycobacterial activity potential of this class of compounds, some of the compounds synthesized were checked against* Mycobacterium tuberculosis* (H37 RV strain) ATCC number 27294. All the compounds were screened for antituberculosis activities and results are worthy of further investigations. The incorporation of Zn^2+^ and Cu^2+^ effectively increases the conformational rigidity of the hydrazones and enhances the fluorescence intensities of the complex which shows that it is good material in photochemical applications of these complexes.

## Supplementary Material

D The material and methods utilised for the synthesis of hydrazone ligands and metal complexes provided in supplementary material. Structures of synthesized compounds and scanned images of spectras (*1*H, *13*C NMR, FTIR, MASS) have been provided in supplementary material.

## Figures and Tables

**Table 1:** Structures of hydrazones 2a–2e (see Figure 10).

Entry	Hydrazide (1)	Hydrazone (2)	Yield (%)	Colour
a	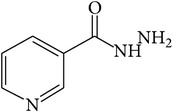	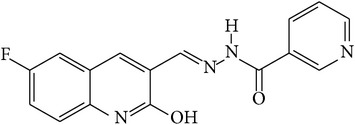	84	Dark yellow

b	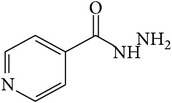	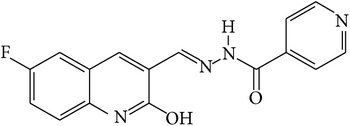	87	Yellow

c	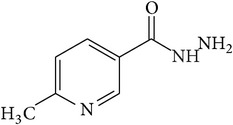	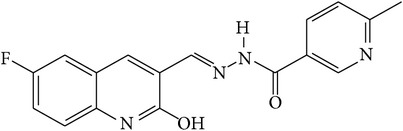	76	Faint yellow

d	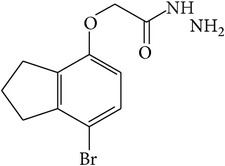	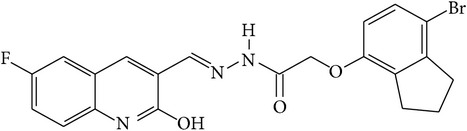	90	Pale yellow

e	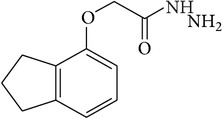	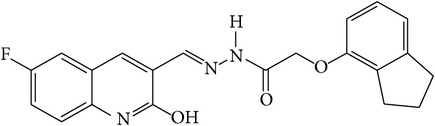	84	Pale yellow

**Table 2:** Proposed structures of Zn(II) and Cu(II) complexes from hydrazones 2a–2e (see Figure 11).

Entry	Metal complex	Yield (%)	Colour
3a	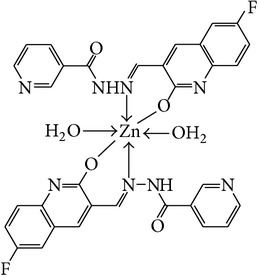	92	Bright yellow

3b	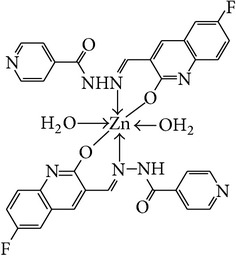	90	Dark yellow

3c	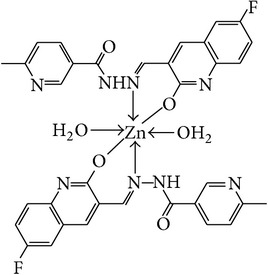	93	Dark yellow

3d	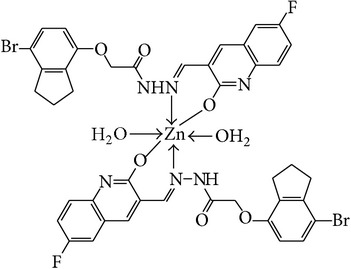	88	Shiny yellow

3e	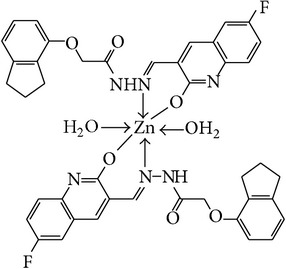	92	Dark yellow

3f	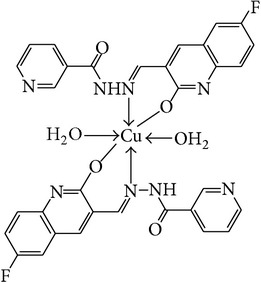	90	Dark green

3g	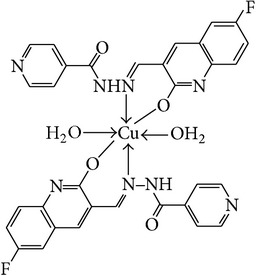	87	Green

3h	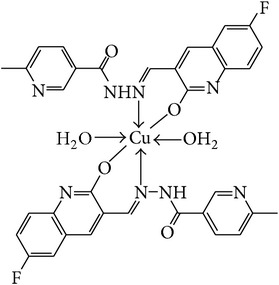	89	Green

3i	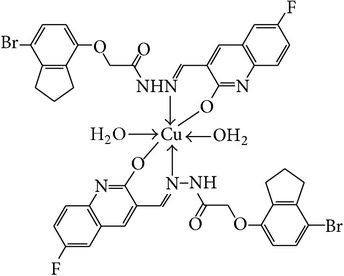	86	Green

3j	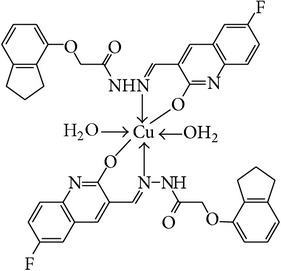	91	Dark green

**Table 3:** ^1^H NMR signals for hydrazones (2a–2e) and their assignments.

Hydrazones	Amidic -NH^*∗*^-	Phenolic -OH^*∗*^	Azomethine -CH^*∗*^=N-
2a	12.16	12.09	9.08
2b	12.26	12.13	8.77
2c	12.11	12.11	8.97
2d	12.13	11.82	8.51
2e	12.10	11.76	8.42

**Table 4:** ^13^C NMR for hydrazones (2a–2e) and their assignments.

Hydrazones	-C^*∗*^=O amide	-C^*∗*^-OH phenolic	-C^*∗*^H=N- azomethine
2a	163.01	161.63	144.63
2b	164.80	162.52	140.97
2c	165.31	160.40	141.29
2d	168.50	162.75	143.72
2e	168.40	161.96	146.95

**Table 5:** FTIR bands for hydrazones (2a–2e) and their assignments.

Compounds	Phenolic *υ*_(OH)_ Cm^−1^	Amide *υ*_(C=O)_ Cm^−1^	Imine *υ*_(C=N)_ Cm^−1^	Phenolic *υ*_(C-O)_ Cm^−1^	*υ*_(C-F)_ Cm^−1^
2a	3208	1660	1625	1425	1294
2b	3488	1650	1630	1427	1288
2c	3444	1662	1628	1438	1234
2d	3538	1656	1627	1425	1263
2e	3193	1668	1625	1428	1232

**Table 6:** FTIR bands for metal complexes (3a–3j) and their assignments.

Complex	Lattice water *υ*_(OH)_ Cm^−1^	Amide *υ*_(C=O)_ Cm^−1^	Imine *υ*_(C=N)_ Cm^−1^	Phenolic *υ*_(C-O)_ Cm^−1^	*υ*_(M-N)_ Cm^−1^	*υ*_(M-O)_ Cm^−1^
3a	3399	1650	1631	1376	574	474
3b	3372	1646	1614	1369	590	449
3c	3392	1652	1619	1380	599	482
3d	3369	1650	1631	1376	599	468
3e	3392	1621	1589	1425	592	470
3f	3436	1665	1616	1380	597	470
3g	3357	1610	1558	1375	501	460
3h	3432	1658	1616	1386	566	474
3i	3426	1658	1617	1382	530	453
3j	3368	1663	1616	1380	599	472

**Table 7:** Molar conductance (Λ*m*) of Cu(II) and Zn(II) complexes.

	Complexes	Molar conductance Λ*m* (Ω^−1^ mol^−1^ cm^2^)
Zn complexes	3a	3.6
	3b	4.9
	3c	3.2
	3d	2.9
	3e	5.3

Cu complexes	3f	7.30
	3g	4.83
	3h	6.42
	3i	4.74
	3j	3.32

**Table 8:** Percentage content of Zn and Cu in metal complexes 3a–3j.

Zn(II) complexes	% Zn content observed (calculated)
3a	9.21 (9.08)
3b	8.96 (9.08)
3c	8.63 (8.74)
3d	6.32 (6.44)
3e	7.80 (7.62)

Cu(II) complexes	% Cu content observed (calculated)

3f	8.73 (8.85)
3g	8.93 (8.85)
3h	8.64 (8.52)
3i	6.31 (6.27)
3j	7.40 (7.42)

**Table 9:** Showing comparative antituberculosis screening results by MIC method.

	Test samples	Sample concentration in *μ*g/mL (MIC)
Hydrazones	2a	50
	2b	25
	2c	50
	2d	50
	2e	25

Zn complexes	3a	25
	3b	25
	3c	12.5
	3d	25
	3e	12.5

Cu complexes	3f	6.25
	3g	12.5
	3h	6.25
	3i	12.5
	3j	25

**Table 10:** The absorption and emission wavelength with intensity.

Compound	Absorption *λ*_max_ (intensity)	Emission *λ*_max_ (intensity)
2a	383 (1.73)	456 (200.11)
2b	388 (2.37)	452 (222.03)
2c	385 (1.42)	459 (203.63)
2d	382 (2.78)	458 (110.40)
2e	381 (1.65)	451 (400.14)
3a	405 (1.49)	479 (791.91)
3b	408 (0.63)	485 (654.31)
3c	405 (2.75)	485 (766.40)
3d	391 (1.50)	466 (453.99)
3e	392 (1.69)	464 (409.44)
3f	401 (0.99)	473 (138.56)
3g	402 (0.69)	464 (284.08)
3h	394 (1.07)	460 (359.55)
3i	399 (0.34)	466 (446.55)
3j	390 (1.87)	456 (704.06)
