# Supplementary material for: Synthesis and Biological Evaluation of New Hydrazone Derivatives of Quinoline and Their Cu(II) and Zn(II) Complexes against Mycobacterium tuberculosis
Source: Bioinorg Chem Appl. 2015 Nov 25;2015:153015. doi: 10.1155/2015/153015 (PMC4673354; doi:10.1155/2015/153015)

SYNTHESIS AND BIOLOGICAL EVALUATION OF NEW HYDRAZONE DERIVATIVES OF QUINOLINE AND THEIR Cu (II) AND Zn (II) COMPLEXES AGAINST MYCOBACTERIUM TUBERCULOSIS

Mustapha C. Mandewale*[1], Bapu Thorat[1], Dnyaneshwar Shelke[1] and Ramesh Yamgar[2]

[1]P.G. and Research Centre, Department of Chemistry, Government of Maharashtra, Ismail Yusuf College of Arts, Science and Commerce, Jogeshwari (E), Mumbai-400 060, India.

[2]Department of Chemistry, Chikitsak Samuha's Patkar-Varde College of Arts, Science and Commerce, Goregaon (W),Mumbai 400 062, India.

Supporting Information

Sr. No.	Table of contents…………………………………………………………………	Page No.
01	Material and Methods	S1-S7
02	SAR Study	S8-S16
03	Biological assay	S17-S21
04	Copies of FTIR, ^{1}H NMR, ^{13}C NMR and Mass spectra of products 2a-2e	S22-S44
05	Copies of FTIR Spectra of products 3a-3j	S45-S54

1. Material and Methods:

All solvents were distilled prior to use. Crude products were purified by column chromatography on silica gel of 60–120 or 100–200 mesh. Thin layer chromatography plates were visualized by exposure to ultraviolet light. IR spectra were recorded on FTIR-7600 Lambda Scientific Pty. Ltd. using KBr pellets. ^{1}H NMR spectra were recorded in DMSO-d_6 solvent on Varian-NMR-Mercury 300 MHz instrument. Chemical shifts (δ) were reported in parts per million (ppm) with respect to TMS as an internal standard. The UV–Visible absorption spectra were recorded with UV spectrophotometer model Shimadzu UV-1800. The path length of the measurements was 1 cm. The fluorescence study was done on a Spectrofluorophotometer model Shimadzu RF-5301pc having 1 cm path length.

Procedure synthesis of hydrazones from 6-Fluoro-2-hydroxyquinoline-3-carbaldehyde [2a-2e]

The 6-Fluoro-2-hydroxyquinoline-3-carbaldehyde (0.200 g, 0.0015 mole) was dissolved in 5 mL ethanol and compound 1a-1e (0.0015 mole) was added. A drop of glacial Acetic acid was added as a catalyst for the reaction. The reaction mixture was refluxed for half an hour. The reaction mixture was cooled in ice bath and precipitated product was filtered. The product was then dried in oven.

Preparation of N'-[(E)-(6-fluoro-2-hydroxyquinolin-3-yl)methylidene]pyridine-3-carbohydrazide [2a]

M.P.: 293-295^{0}C; UV λmax: 383 nm; MS [M+H]: 311.59; FTIR(KBr cm^{-1}): 3208 (Phenolic–OH), 3073 (-N-H amide), 1660 (azomethine –CH=N-), 1625 (C=O amide), 1425 (phenolic C-O), 1294 (C-F quinoline); ^{1}H NMR (300 MHz, DMSO-d_6) δ: 7.35-7,42(m, 2H), 7.55(s,1H), 7.75-7.78(d, 1H), 8.25-8.28(d, 1H), 8.49(s,1H), 8.69-8.75(m,2H), 9.08(s,1H), 12.09(s,1H), 12.16(s,1H); ^{13}C NMR (75MHz, DMSO-d_6) δ: 163.01 (-C=O amide), 161.63(-C-O phenolic), 150.02(-C-F quinoline), 148.75, 148.24, 147.26, 146.23(-C=N- azomethine), 133.28, 130.99, 130.66, 130.50, 127.90, 126.63, 124.99, 119.43, 110.5; Elemental analysis: observed (calculated): C 61.97% (61.93%), H 3.66% (3.57%), N 18.20% (18.06%).

Preparation of N'-[(E)-(6-fluoro-2-hydroxyquinolin-3-yl)methylidene]pyridine-4-carbohydrazide [2b]

M.P.: >300^{0}C; UV λmax: 388 nm; MS [M-H]: 309.27; FTIR(KBr cm^{-1}): 3488 (phenolic–OH), 3153 (N-H amide), 3025 (aromatic C-H), 1650 (imine –CH=N-), 1630 (C=O amide), 1427 (phenolic C-O), 1288 (C-F quinoline); ^{1}H NMR (300 MHz, DMSO-d_6) δ: 7.32 – 7.45 (m, 2H), 7.77 – 7.85 (m, 3H), 8.50 (s, 1H), 8.71(m, 2H), 8.77(s, 1H), 12.13 (s, 1H), 12.26 (s, 1H); ^{13}C NMR (75MHz, DMSO-d_6) δ: 164.8(-C=O amide), 162.52(-C-O phenolic), 152.32(C-F quinoline), 150.01, 147.26, 140.97(-C=N- azomehine), 134.57, 131.02, 131.52, 127.43, 125.71, 119.46, 118.21, 110.5; Elemental analysis: observed (calculated): C 61.98% (61.93%), H 3.72% (3.57%), N 18.17% (18.06%).

Preparation of N'-[(E)-(6-fluoro-2-hydroxyquinolin-3-yl)methylidene]-6-methylpyridine-3-carbohydrazide [2c]

M.P.: >300^{0}C; UV λmax: 385 nm; MS [M+H]: 325.16; FTIR(KBr cm^{-1}): 3444 (phenolic–OH), 3228 (N-H amide), 2933 (aromatic C-H), 2886 (aliphatic C-H), 1662 (imine –CH=N-), 1628 (C=O amide), 1438 (phenolic C-O), 1234 (C-F quinoline); ^{1}H NMR (300 MHz, DMSO-d_6) δ: 2.54(s,3H), 7.36-7.43(m,3H), 7.76-7.79(m,1H), 8.17-8.19(m,1H), 8.49(s,1H), 8.70(s,1H), 8.97(s,1H), 12.16(s,2H); ^{13}C NMR (75MHz, DMSO-d_6) δ: 163.31(-C=O amide), 160.40(-C-O- phenolic), 155.47(-C-F quinoline), 158.48, 147.94, 145.66, 141.29(-C=N- azomethine), 131.29, 131.06, 130.01, 128.69, 126.72, 125.23, 122.08, 117.93, 112.4, 23.97(-CH_3 pyridine); Elemental analysis: observed (calculated): C 62.87% (62.96%), H 4.13% (4.04%), N17.34% (17.28%).

Preparation of 2-[(7-bromo-2,3-dihydro-1*H*-inden-4-yl)oxy]-*N*'-[(*E*)-(6-fluoro-2-hydroxyquinolin-3-yl)methylidene]acetohydrazide [2d]

M.P.: >300^{0}C; UV λmax: 382 nm; MS [M+H]: 458.00; FTIR(KBr cm^{-1}): 3538 (phenolic–OH), 3432 (N-H amide), 3002 (aromatic C-H), 2848 (aliphatic C-H), 1656 (imine –CH=N-), 1627 (C=O amide), 1425 (phenolic C-O), 1263 (C-F quinoline); ^{1}H NMR (300 MHz, DMSO-d_6) δ: 2.85-2.97(m,6H), 4.48(s,2H), 7.24-7.47(m,2H), 7.59-7.62(m,1H), 7.74-7.81(m,1H), 8.23(s,1H), 8.42(s,1H), 8.51(s,1H), 11.82(s,1H), 12.13(s,1H); ^{13}C NMR (75MHz, DMSO-d_6) δ: 168.50(-C=O amide), 162.75 (C-O- phenolic), 159.14(C-F quinoline), 155.80, 154.55, 146.26, 143.72(-C=N- azomehine), 135.14, 133.59, 131.79, 130.06, 127.43, 125.93, 122.20(-C-Br), 120.93, 111.97, 109.95, 67.83(-CH_2-O-), 29.83(CH_2 aliphatic), 29.77(CH_2 aliphatic), 25.78(CH_2 aliphatic); Elemental analysis: observed (calculated): C 55.50% (55.04%), H 3.69% (3.74%), N 9.20% (9.17%).

Preparation of 2-(2,3-dihydro-1*H*-inden-4-yloxy)-*N*'-[(*E*)-(6-fluoro-2-hydroxyquinolin -3-yl)methylidene]acetohydrazide [2e]

M.P.: >300^{0}C; UV λmax: 381 nm; MS [M+H]: 380.44; FTIR(KBr cm^{-1}): 3193 (phenolic–OH), 3064 (N-H amide), 2950 (aromatic C-H), 2854 (aliphatic C-H), 1668 (imine –CH=N-), 1625 (C=O amide), 1428 (phenolic C-O), 1232 (C-F quinoline); ^{1}H NMR (300 MHz, DMSO-d_6) δ: 1.99(m,2H), 2.83(m,4H), 4.65(s,2H), 6.60-6.65(m,1H), 6.82(m,1H), 7.05(m,1H), 7.33-7.42(m,2H), 7.60-7.62(m,1H), 8.22(s,1H), 8.42(s,1H), 11.76(s,1H), 12.10(s,1H); ^{13}C NMR (75MHz, DMSO-d_6) δ: 168.40(-C=O amide), 162.96(-C-O- phenolic), 159.81(-C-F quinoline), 154.90, 147.35, 146.95(-C=N- azomethine), 137.5, 135.14, 130.99, 130.8, 130.66, 127.90, 126.63, 125.01, 119.43, 113.72, 110.5, 66.86, 32.61(CH_2 aliphatic), 29.67(CH_2 aliphatic), 25.38(CH_2 aliphatic); Elemental analysis: observed (calculated): C 66.52% (66.48%), H 4.70% (4.78%), N 11.21% (11.08%).

<u>Procedure for synthesis of Zn^{2+} and Cu^{2+} complexes [3a-j]</u>

The solution of metal salt [$ZnCl_2$, $CuCl_2$] dissolved in ethanol was added gradually to a stirred ethanolic solution of the Schiff base hydrazones [2a-e], in the molar ratio 1:2. The reaction mixture was further stirred for 2–4 h at 60^0 C. Then it was cooled in ice bath to ensure the complete precipitation of the formed complexes. The precipitated solid complexes were filtered and washed four times with water. Finally, the complexes were washed with diethyl ether and dried in vacuum desiccators over anhydrous $CaCl_2$.

<u>Elemental Analysis of metal complexes:</u>

The quantitative estimation of Zn^{2+} and Cu^{2+} has been done by complexometric titration with standard EDTA solution. In a titration an accurately known mass of metal complex is dissolved in an aqueous solution by chemical treatment such as acid-digestion of solid metal complex samples and diluted with high purity water to an accurately known volume. Then an accurately known volume of the aliquot is pipetted into a titration vessel and the analyte of interest is carefully titrated with a standardized EDTA solution to the endpoint of the titration.

Estimation of Zinc

Experimental Procedure:

1. Weigh accurately approximately 0.1 g samples of the Zinc complexes (2a-2e).
2. Place copper complex in porcelain dish and add concentrated 5 mL H_2SO_4. Heat on sand bath near dryness. Repeat it for 2 times more. Then add 5 mL of concentrated HCL and heat again to get Zn (II) in chloride (water soluble) form. Heat this nearly to dryness and extract with deionized water.
3. Carefully dilute sample solution in the 250 mL volumetric flask to the mark with deionized water. Mix it thoroughly.
4. Pipette out 25 mL aliquot into conical flask. Add 15 mL of deionized water, 9-10 mL of pH 10 buffer, and 3 drops of Eriochrome Black T immediately prior to titrating a sample. Titrate with standardized EDTA until the pink solution turns light blue.
5. Calculate the milligrams of zinc in the total sample. Remember that each aliquot represents one tenth of the total sample volume of a 25 mL aliquot titrated out of 250 mL total volume.

CALCULATIONS:

The molarity of the Zn^{2+} standard solution (M_{Zn}) is calculated in normal fashion using the molar mass of Zinc Chloride weighed out and the total volume in liters of the standard solution prepared.

$$Mzn = \frac{Moles\ of\ Zn(II)}{Volume\ of\ solution\ in\ mL} = \frac{\frac{mass\ of\ Zn}{molar\ mass\ of\ ZnCl2}}{Volume\ of\ solution\ in\ mL}$$

Calculate the molarity of the EDTA from the volume of EDTA used in the titration of each aliquot of the Zn^{2+} standard solution and the known 1:1 stoichiometry between Zn and EDTA in the reaction. If the reaction has 1:1 stoichiometry, then

mmol EDTA = mmol Zn

The mmol of each constituent is obtained by multiplying the molarity of each of the two solutions times the volume in mL of each solution used to reach the endpoint, ep:

$$M\ EDTA \times V\ EDTA = M\ Zn \times V\ Zn$$

The volume of the Zn standard solution originally taken was 25.00 mL and the volume of EDTA used is the volume used to reach the endpoint, V ep = V EDTA, in mL. Therefore,

$$M\ EDTA = (M\ Zn \times V\ Zn\ ) / V\ EDTA = 25.00 \times M\ Zn / V\ EDTA$$

Substituting molarity times the volume of EDTA used in each titration of the Zn unknown produces:

$$mmol\ Zn = mmol\ EDTA = mmol\ EDTA \times V\ EDTA = mmol\ EDTA \times V\ ep$$

The mass of Zn obtained in a single titration, in mg, is equal to the number of mmol of Zn times its molar mass (MM):

$$mgZn = mmolZn \times MMZn = mmolZn \times 65.38\ mg/mmol$$

And the total mass of Zn in the original 250 mL sample is therefore 10 times this amount.

$$\frac{\text{mass Zn(II)}}{\text{mass Zn(II) Complex}} \times 100 = \%\ \text{Zn(II) present in complex}$$

Estimation of Copper

Experimental Procedure:

1. Weigh accurately three approximately 0.1 g samples of the copper complex.
2. Place copper complex in porcelain dish and add concentrated H_2SO_4 5mL. Heat on sand bath near dryness. Repeat for 2 times more. Then add 5 mL of concentrated HCL and heat again to get Cu (II) in chloride (water soluble) form. Heat nearly to dryness and extract with deionized water and dilute to 100 mL in standard volumetric sample. Transfer 25 mL in conical flask.
3. Add three drops of indicator to sample, Titrate each sample with the standardized EDTA. The light yellow solution turns green near the end point, then suddenly purplish blue at the end point.
4. Calculate the milligrams of Copper in the total sample. Remember that each aliquot represents one fourth of the total sample volume of a 25 mL aliquot titrated out of 100 mL total volume.

Calculations:

During the titration, the $EDTA^{2-}$ forms a more stable complex and frees the indicator, which then displays its original color. The appearance of the free indicator means that all metal ions have been complexed by $EDTA^{2-}$, which signals the end point. At the end point, the following equation applies:

$$N_{EDTA} \times V_{EDTA} = N_{Cu\,(II)} \times V_{Cu\,(II)} = \text{meq Cu(II), V is given in mL}$$

$$\text{The mass of Cu (II)} = \text{eq Cu(II))} \times \text{(equivalent mass of Cu(II))}$$

$$\frac{\text{mass Cu(II)}}{\text{mass Cu(II) Complex}} \times 100 = \%\ \text{Cu(II) present in complex}$$

Results:

Zn Complexes	% Zn Observed (Calculated)	Cu Complexes	% Cu Observed (Calculated)
**3a**	9.21 (9.08)	**3f**	8.73 (8.85)
**3b**	8.96 (9.08)	**3g**	8.93 (8.85)
**3c**	8.63 (8.74)	**3h**	8.64 (8.52)
**3d**	6.32 (6.44)	**3i**	6.31 (6.27)
**3e**	7.80 (7.62)	**3j**	7.40 (7.42)

REFERENCE:

D. A. Skoog, D. M. West, F. J. Holler, and S. R. Crouch, *Analytical Chemistry: An Introduction*, 7th ed., Chapter 15, pp. 345-381.

2. SAR Study:

The Cresset's software Forge is a molecular design and SAR (structure activity relationship) interpretation tool that generates and uses molecular alignments as a way to make meaningful comparisons across chemical series. The interaction between a ligand and a protein involves electrostatic fields and surface properties (e.g. hydrogen bonding, hydrophobic surfaces and so on). Two molecules which both bind to a common active site tend to make similar interactions with the protein and hence have highly similar field properties. Accordingly, using these properties to describe molecules is a powerful tool for the medicinal chemist as it concentrates on the aspects of the molecules that are important for biological activity. In Forge, molecules can be aligned by using the Fields of the molecules, by using shape properties or by using a common substructure. Using the Fields gives a 'protein's view' of how the molecules would line up in the active site, generating ideas on how molecules with different structures could interact with the same protein. Using substructure or common shape properties shows how the Fields around a single chemical series varies with activity and in many cases these can be automatically examined to give a 3D quantitative structure active relationship (QSAR) with predictive power for new ideas for synthesis.

Interpretation of Field Point Patterns

Molecules bear different types of field points. Larger field points represent stronger points of potential interaction. Throughout Cresset's software the blue points are negative field points which like to interact with positives/H-bond donors on a protein. Whereas red points are positive field points which like to interact with negatives/H-bond acceptors on a protein (Figure No. 01). Similarly the yellow points are van der Waals surface field points which describing possible surface/ van der Waals interactions. It can be seen that ionic groups give rise to the strongest electrostatic fields. Hydrogen bonding groups also give strong electrostatic fields. Aromatic groups encode both electrostatic and hydrophobic fields. Aliphatic groups such as the methyl or cyclopentyl group give rise to hydrophobic and surface points but are essentially electrostatically neutral (Figure No. 02).

Figure No. 01: Showing details of point force fields.

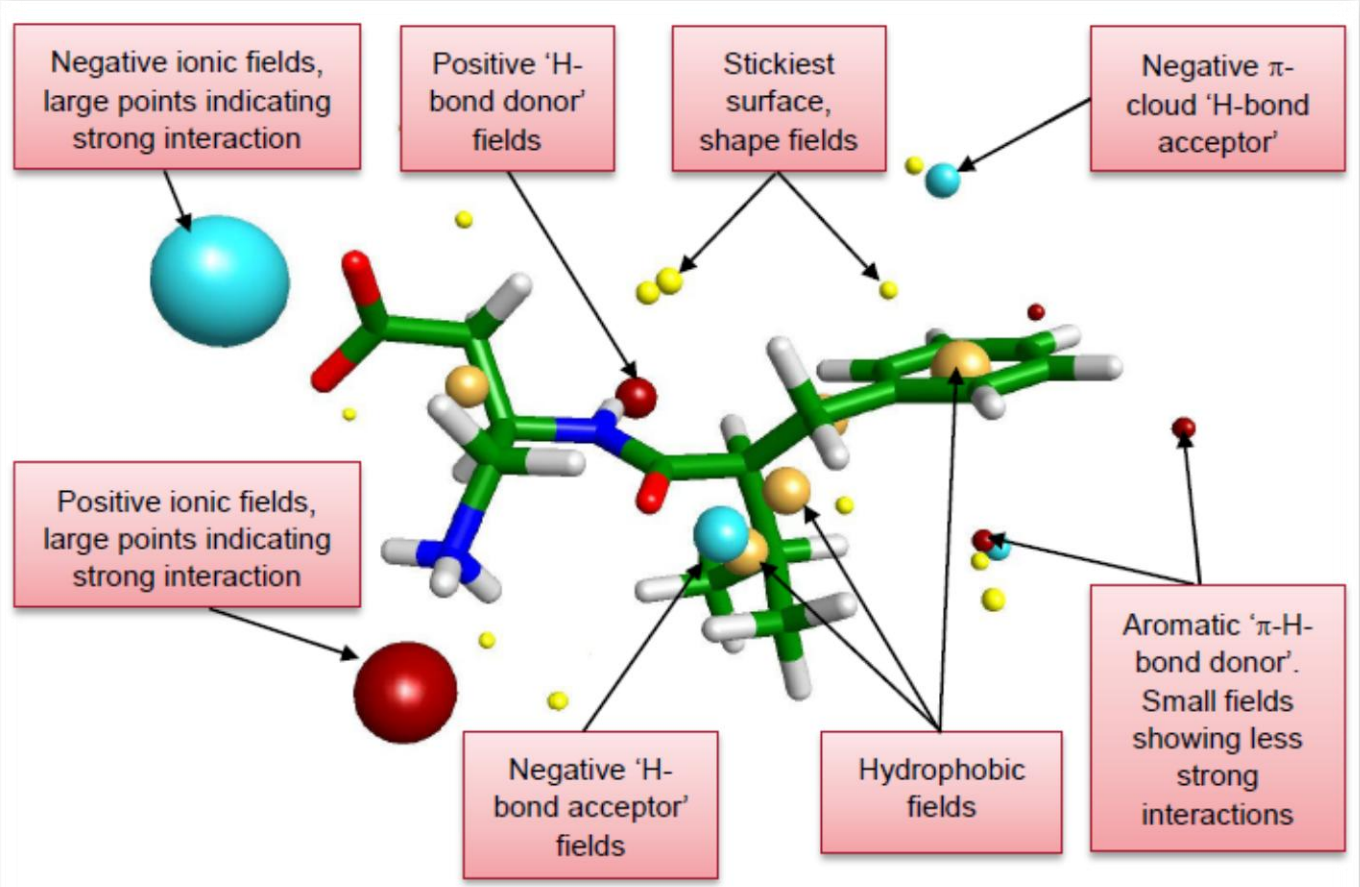

Interpretation of a field point pattern. The size of the point indicates the potential strength of the interaction

Figure No. 02: Showing different electrostatic regions.

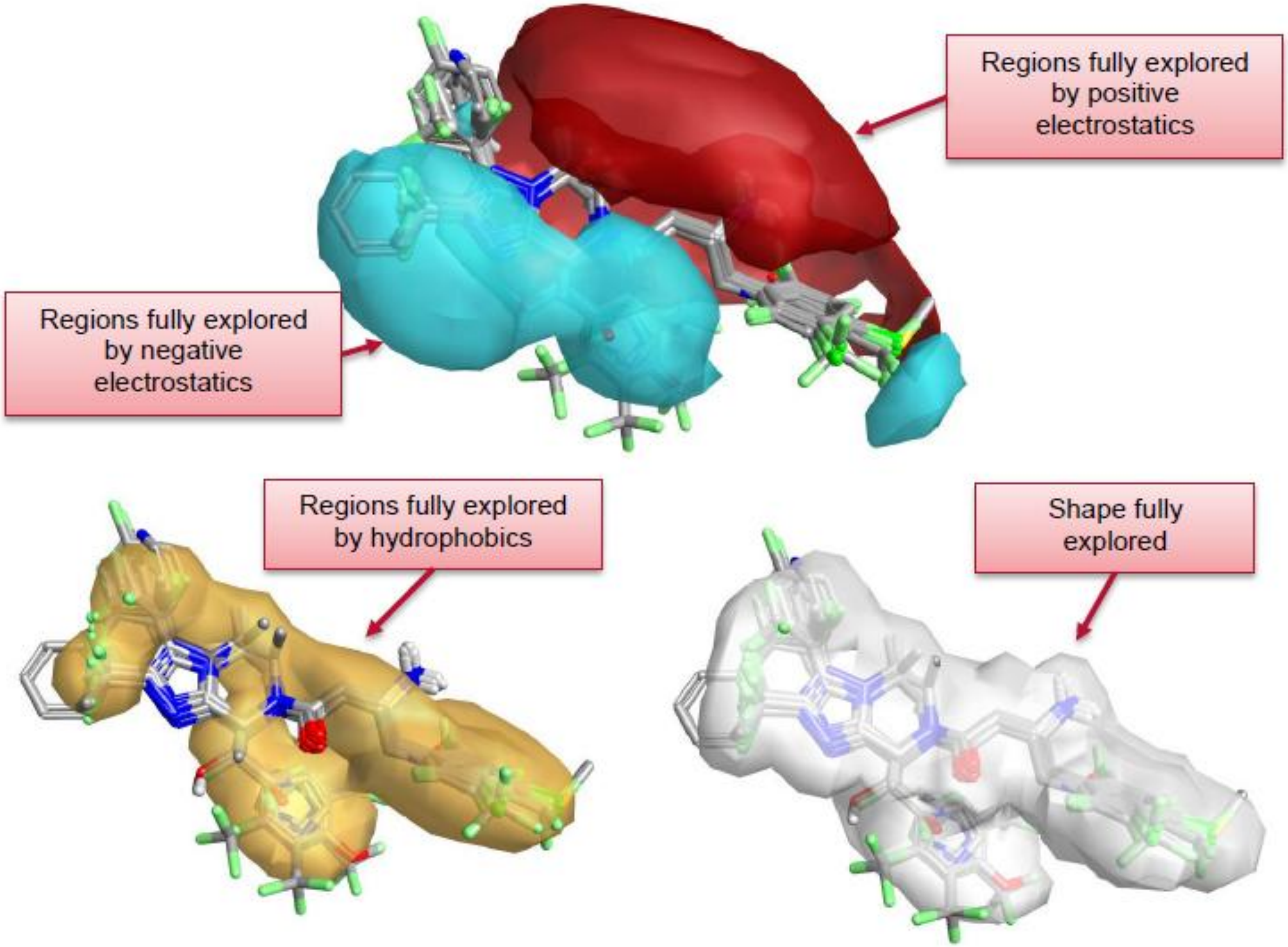

To generate these fields, we use XED (Extended Electron Distribution) molecular mechanics force field, which uses off-atom sites to more accurately describe the electron distribution in a molecule, as opposed to other force fields where charges are placed at the atomic nuclei only.

For SAR study we have selected Ciprofloxacin as reference compound as it show strong anti-tubercular activity against *Mycobacterium tuberculosis*. The bactericidal action of ciprofloxacin results from inhibition of the enzymes *topoisomerase II* (DNA-*gyrase*) and *topoisomerase IV*, which are required for bacterial DNA replication, transcription, repair, strand supercoiling repair, and recombination.

By keeping in the mind two molecules which both bind to a common active site of receptor tend to make similar interactions with the protein and hence have highly similar field properties, we have compared the field properties of the ciprofloxacin with synthesized hydrazone derivatives 2a-2e. This comparison strengthens the correlation between the theoretical and observed activities of the target compounds (Figure No. 03 to 07).

Figure No. 03: Showing different electrostatic regions of the products 2a-2e.

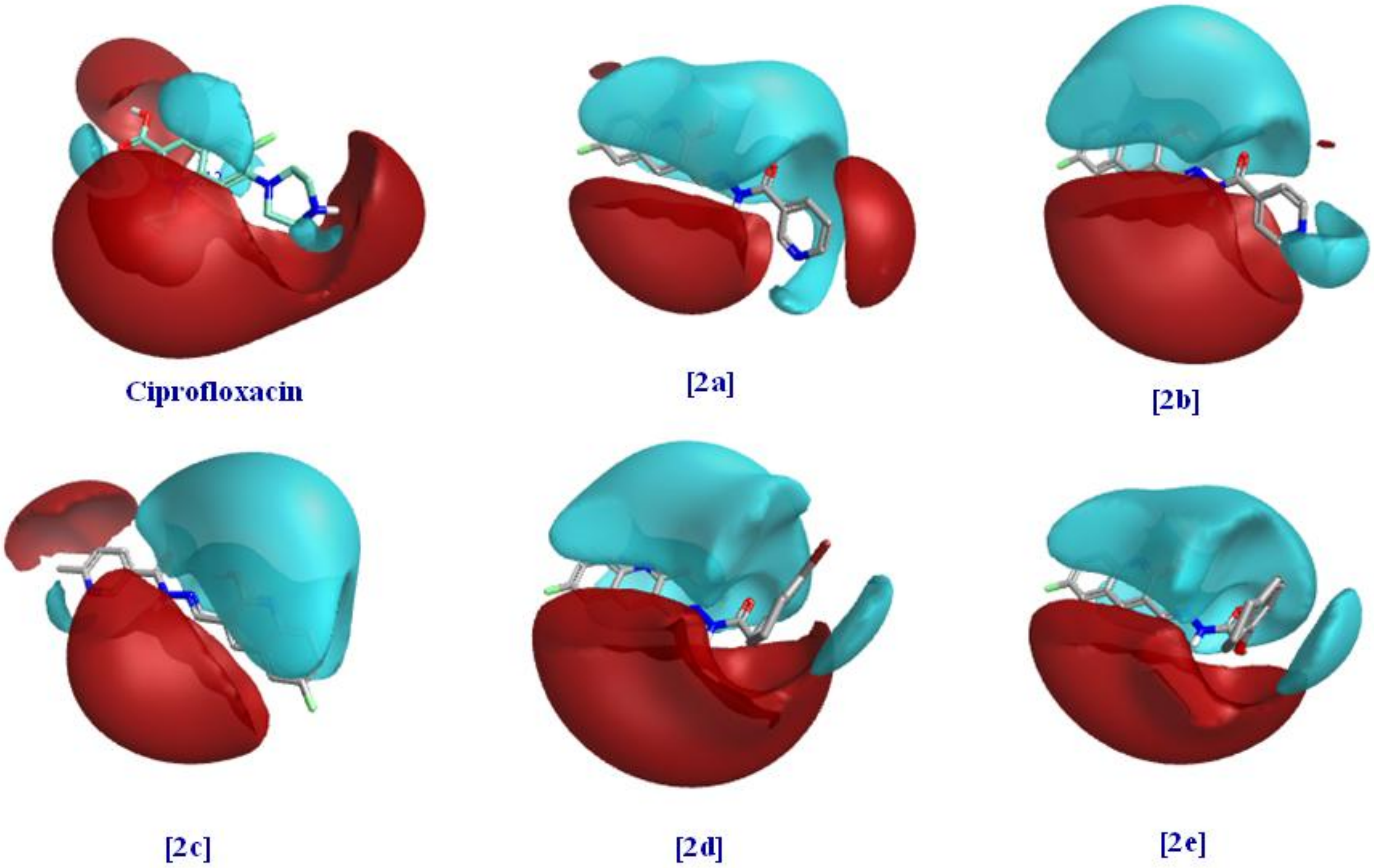

Figure No. 04: Showing point charges of the products 2a-2e.

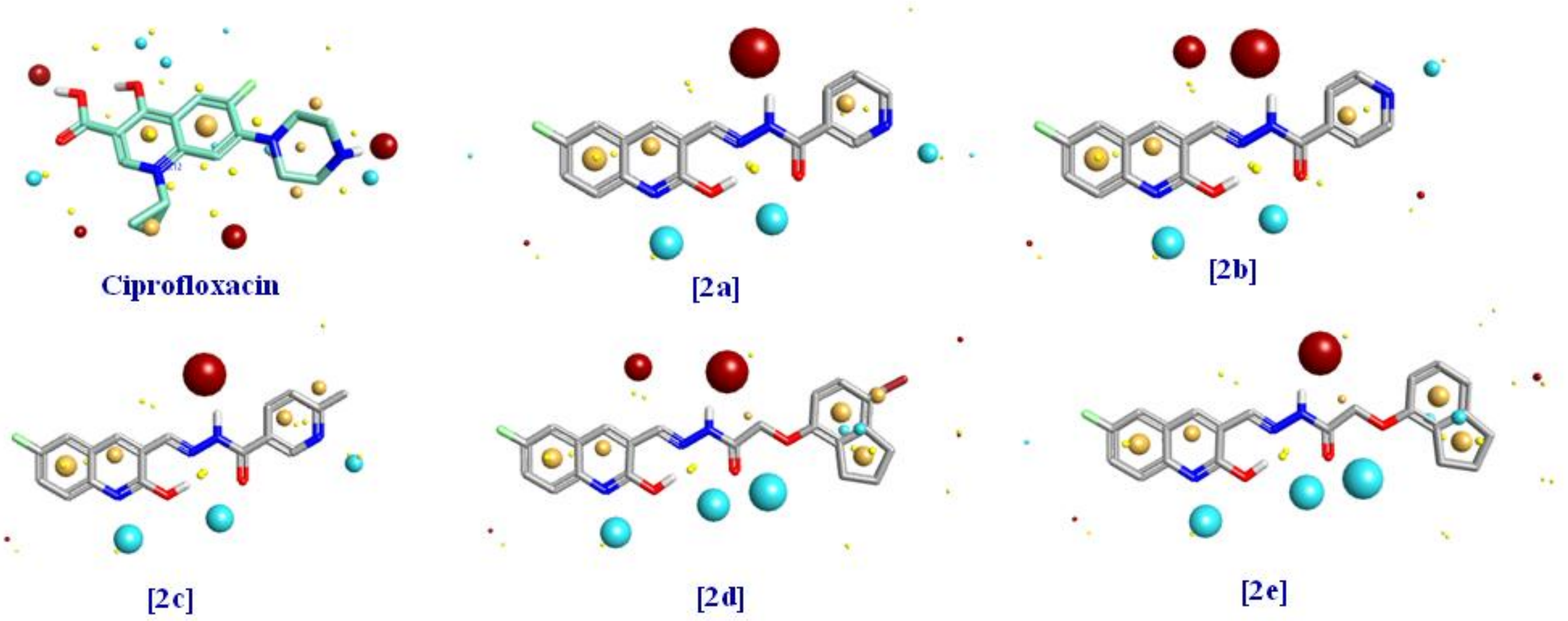

Figure No. 05: Hydrophobic regions for products 2a-2e.

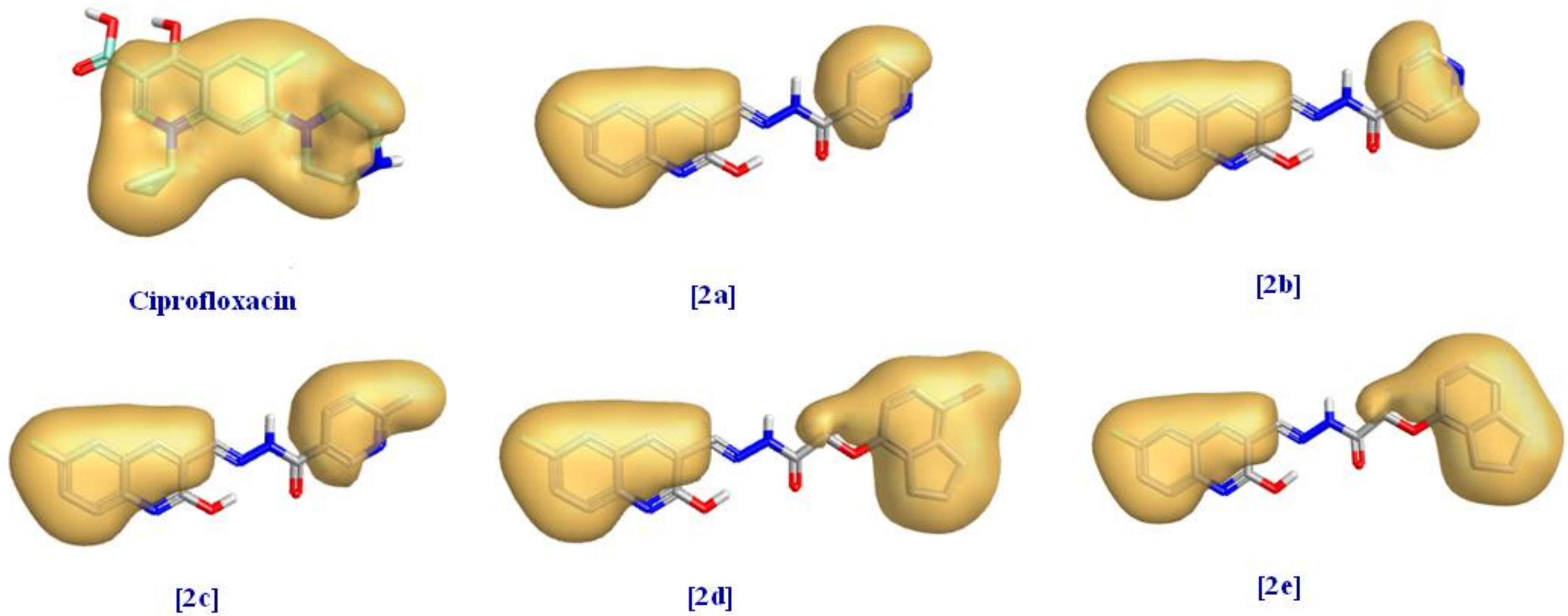

Figure No. 06: Showing positive electrostatic region of products 2a-2b.

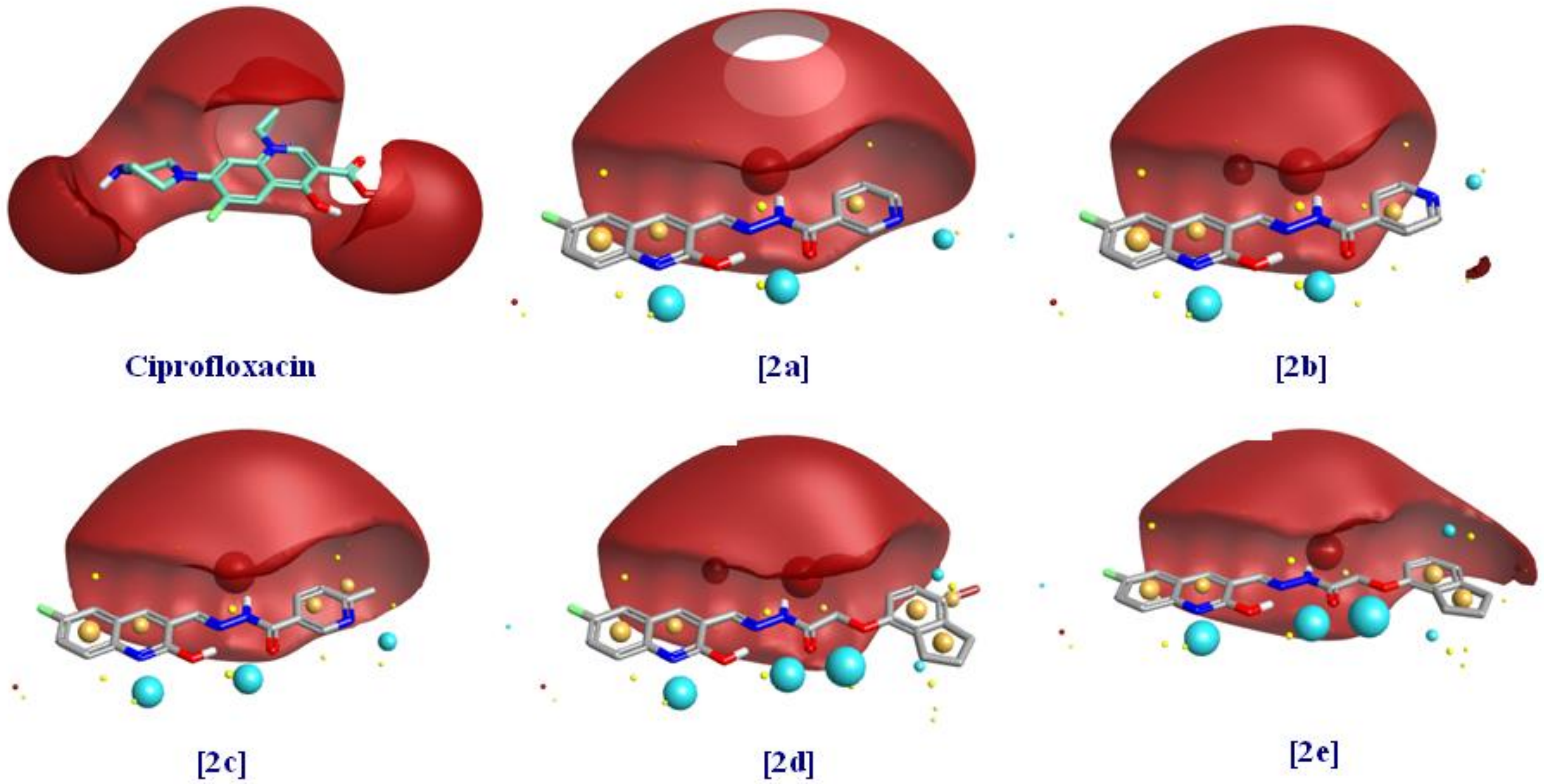

Figure No. 07: Showing negative electrostatic region on products 2a-2e

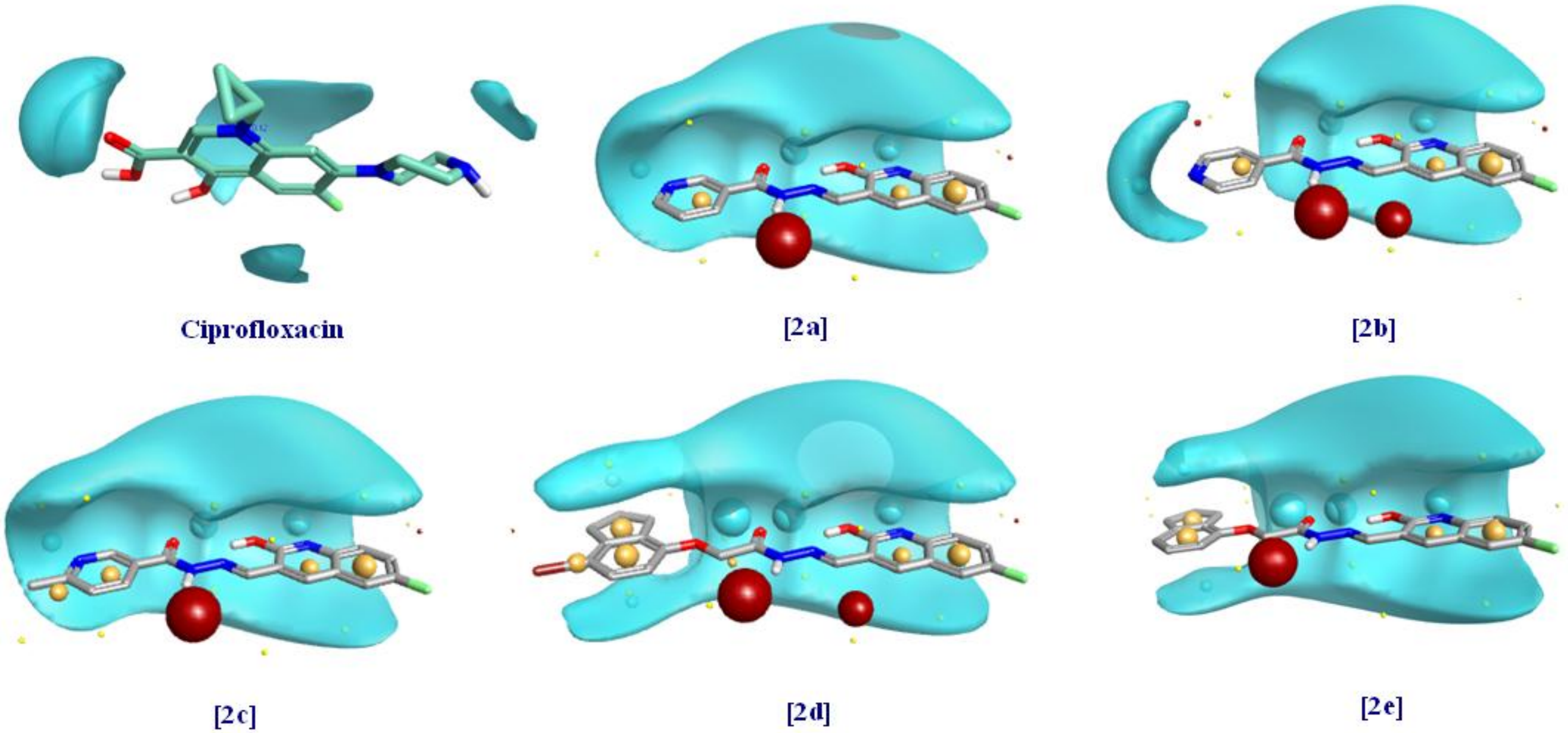

3. Biological Assay

3.1. Antitubercular Studies:

The antitubercular activity of the hydrazone ligands and their metal complexes was tested against *Mycobacterium tuberculosis* (H37 RV strain) ATCC No- 27294 to find out their potency as antimicrobial agent by MIC method (minimum inhibitory concentration).

3.2. Microbiological Method:

The antibacterial activity of hydrazones and their metal complexes were tested against *Mycobacterium tuberculosis* using microplate Alamar Blue assay (MABA). This methodology is non-toxic and reagents used are thermally stable. It also shows good correlation with proportional and BACTEC radiometric method and reproducible results.

To minimize the evaporation of medium in the test wells during incubation 200µl of sterile deionized water was added to all outer perimeter wells of sterile 96 wells plate. The 96 wells plate received 100 µl of the Middlebrook 7H9 broth and serial dilution of compounds were made directly on plate. The concentration of the test sample was prepared between 100 to 0.8 µg/ml ranges. These plates were covered and sealed with paraffin and incubated at 37ºC for five days. Then in next step 25µl of freshly prepared 1:1 mixture of Almar Blue Reagent Tween 10% and 80% was added to the plate and incubated for 24 hrs in incubator. A blue color in the well was indicates bacterial growth whereas pink color show growth of bacteria. From this experiment the MIC can be defined as lowest drug concentration which prevented the color change from blue to pink.

Table No. 01: Showing anti-tuberculosis screening results by MIC method.

	Test Samples	Sample concentration in μg/mL (MIC)
**Hydrazones**	**2a**	**50**
	**2b**	**25**
	**2c**	**50**
	**2d**	**50**
	**2e**	**25**
**Zn Complexes**	3a	**25**
	3b	**25**
	3c	**12.5**
	3d	**25**
	3e	**12.5**
**Cu Complexes**	3f	**6.25**
	3g	**12.5**
	3h	**6.25**
	3i	**12.5**
	3j	**25**

Figure No. 08: Anti-mycobacterium study (A) Standard, (B) Hydrazones 2a-e, (C) Metal complexes 3a-j

[A]

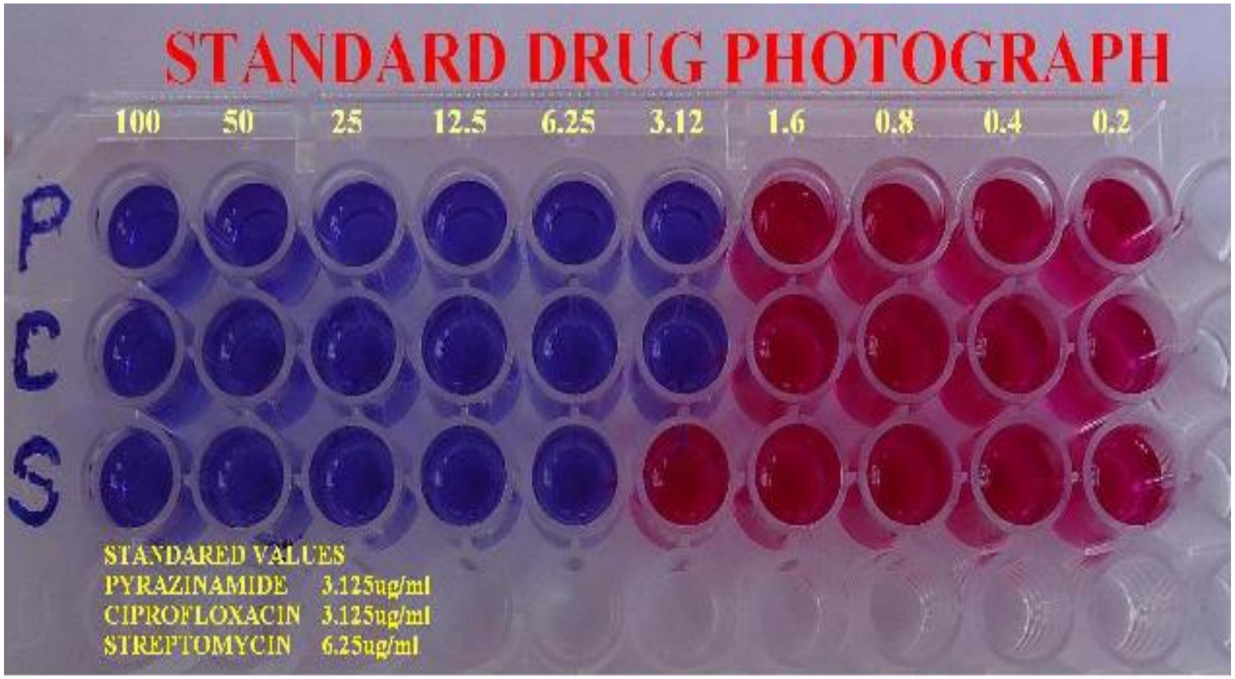

[B]

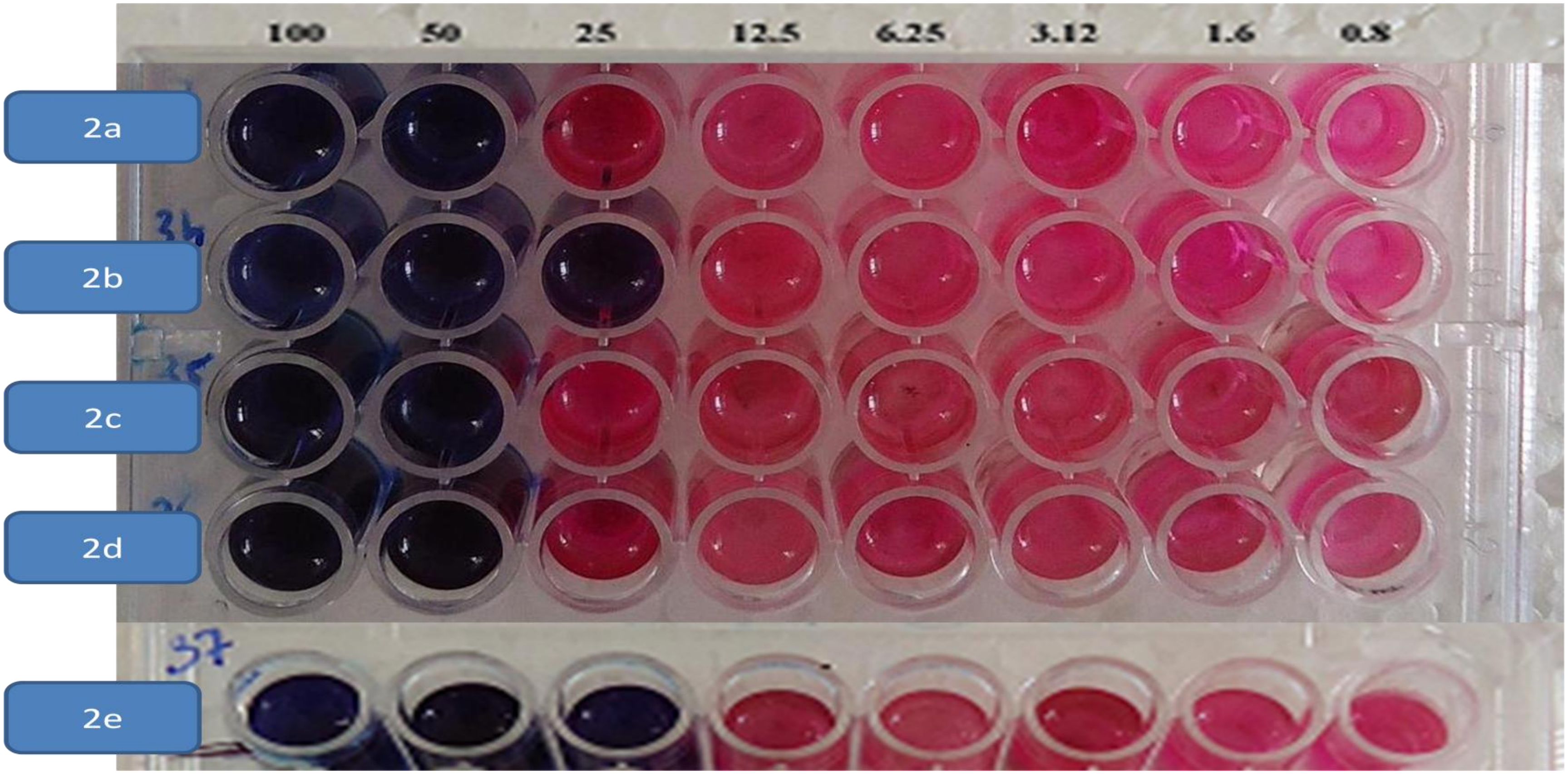

[C]

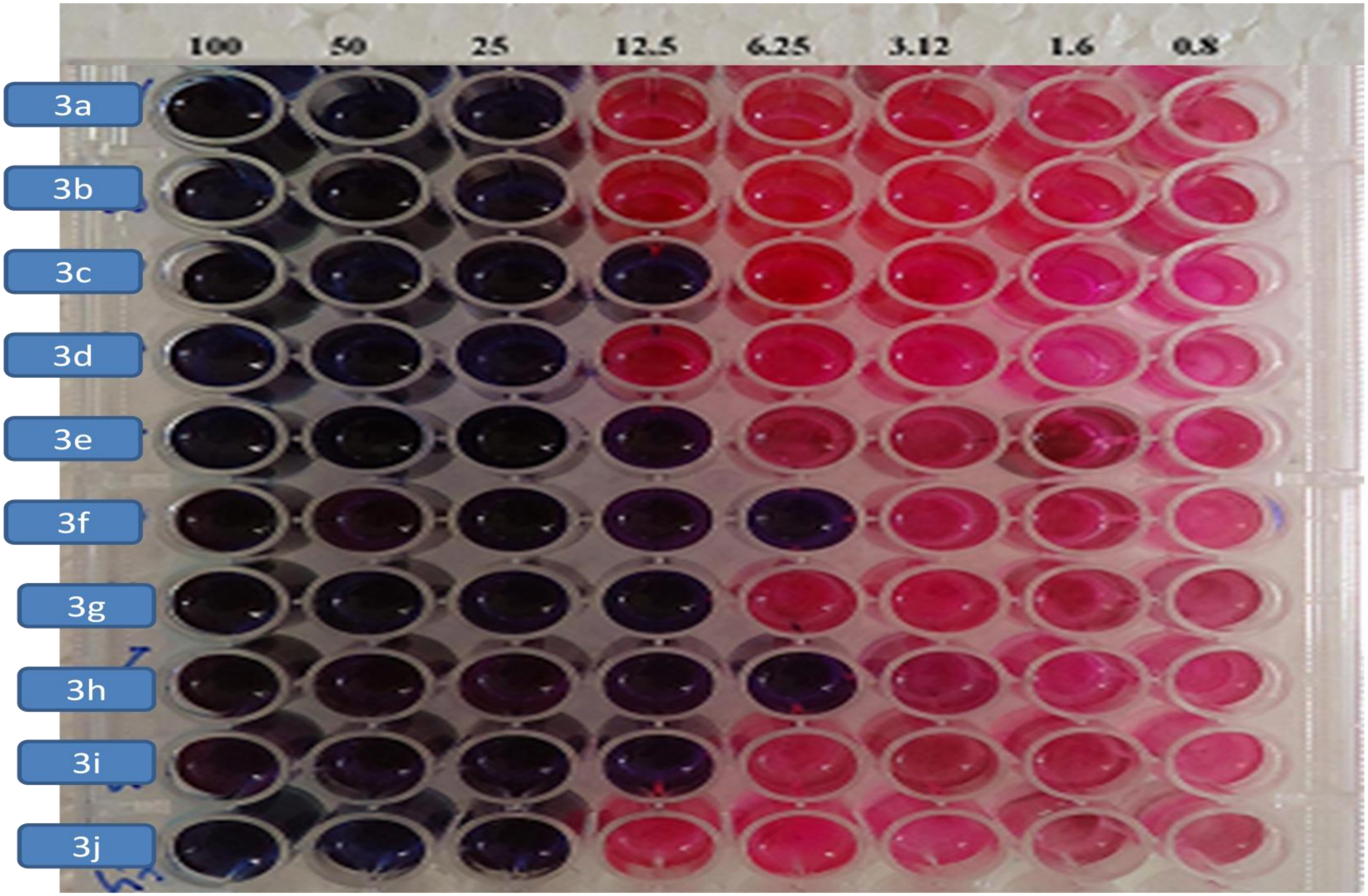

4. Copies of FTIR, 1H NMR, 13C NMR and Mass Spectra of compounds 2a-2e.

<u>FTIR spectra of 6-fluoro-2-hydroxyquinolin.</u>

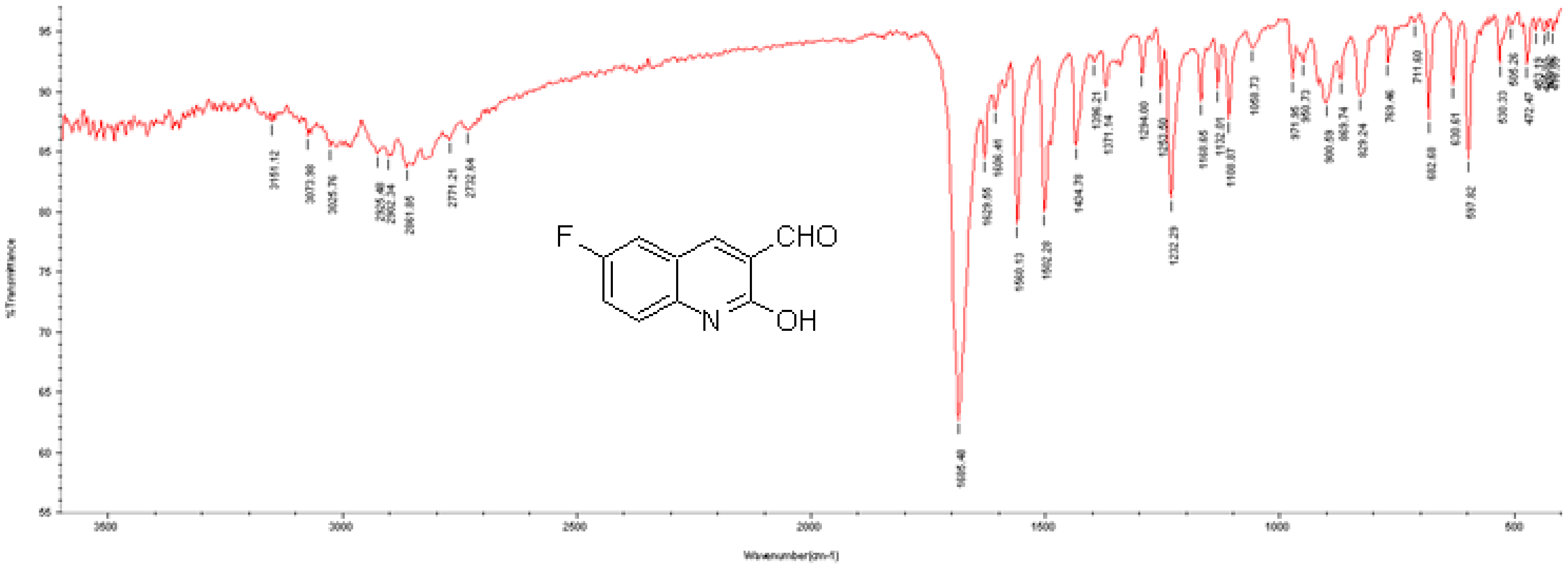

^{1}H NMR Spectra of 6-fluoro-2-hydroxyquinolin.

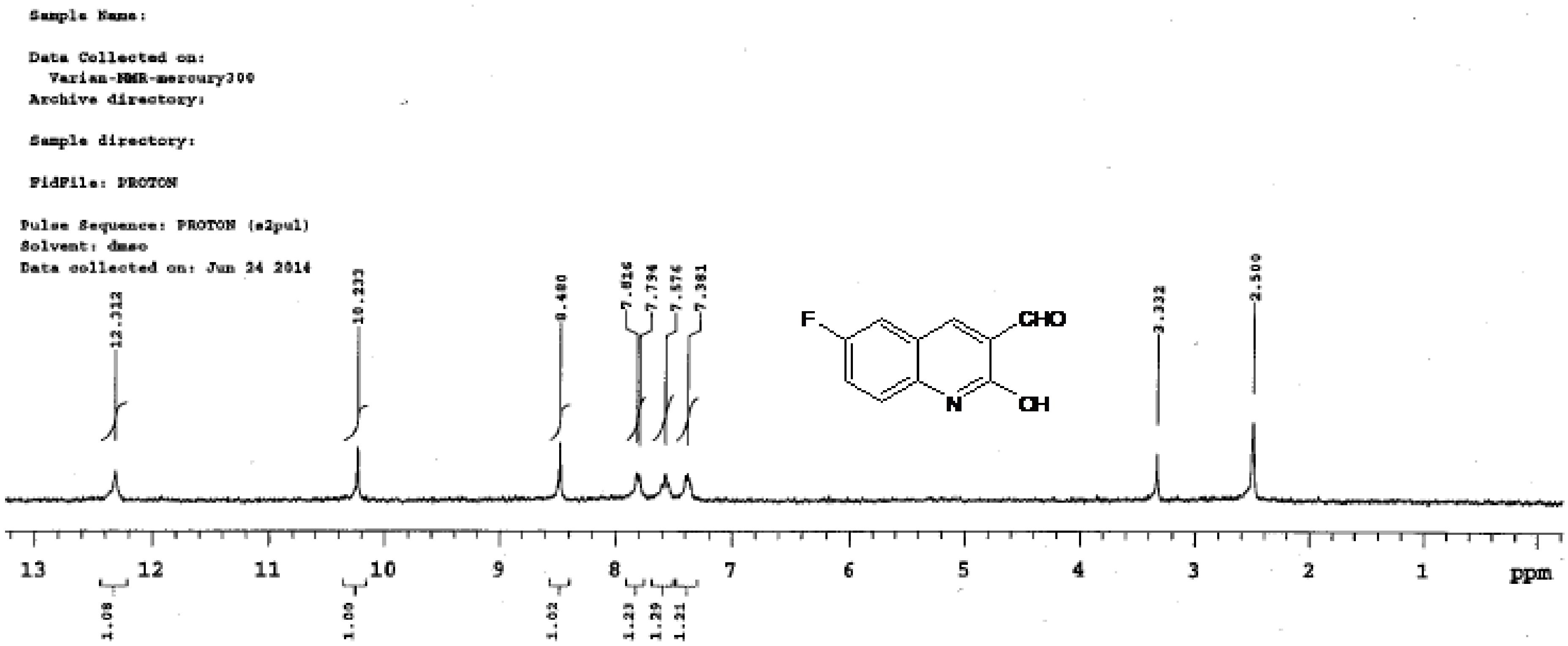

Mass Spectra of 6-fluoro-2-hydroxyquinolin

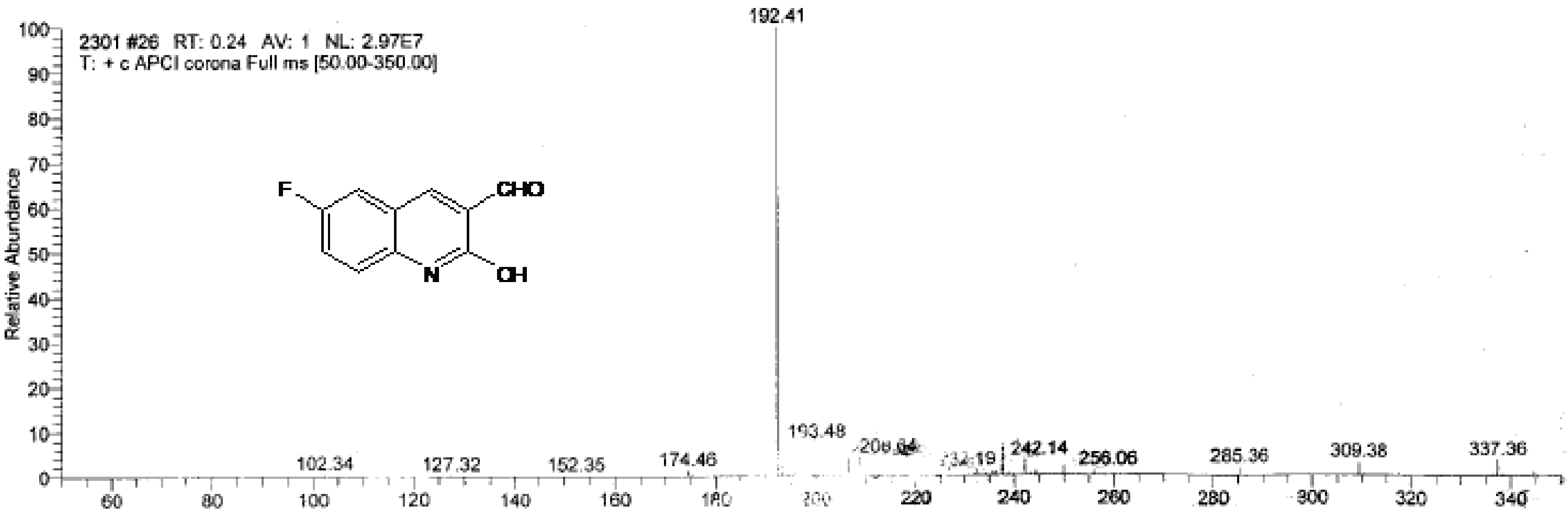

FTIR Spectra of [2a]

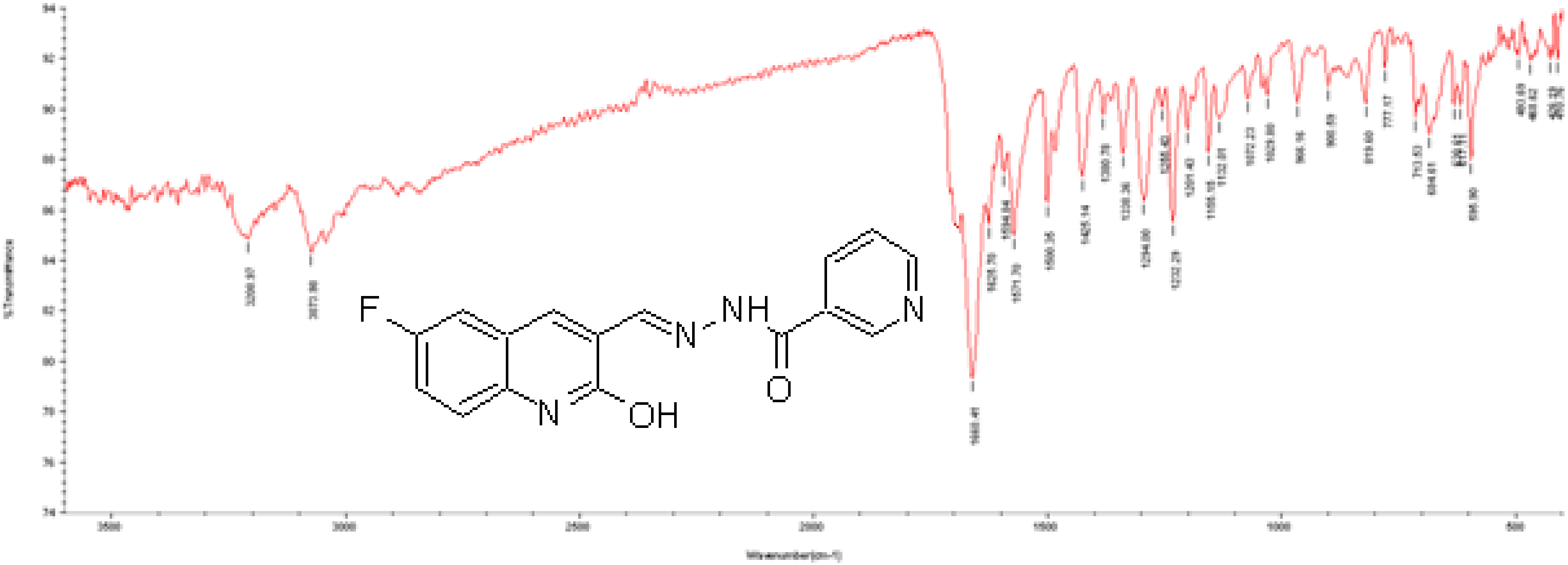

^{1}H NMR Spectra of [2a]

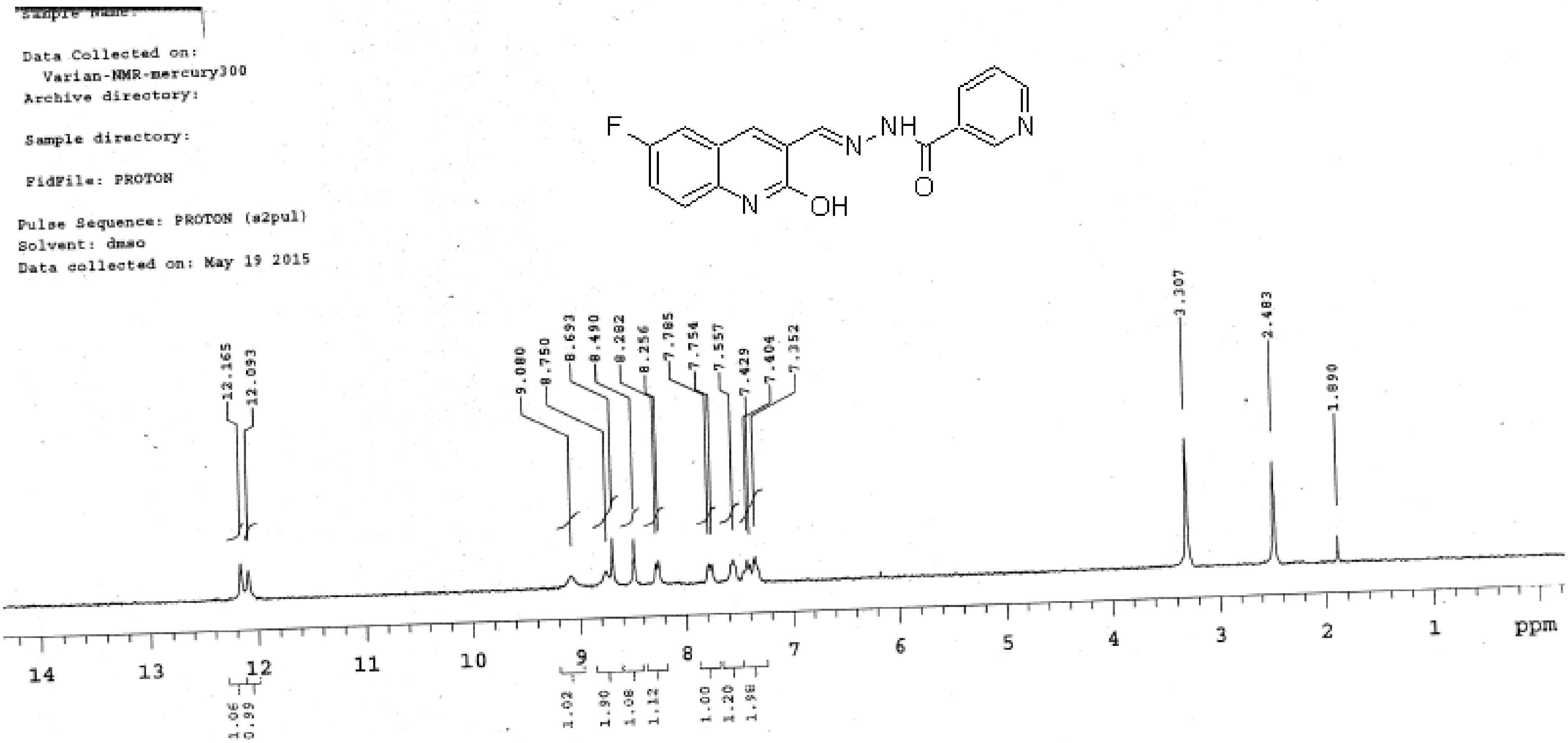

Mass Spectra of [2a]

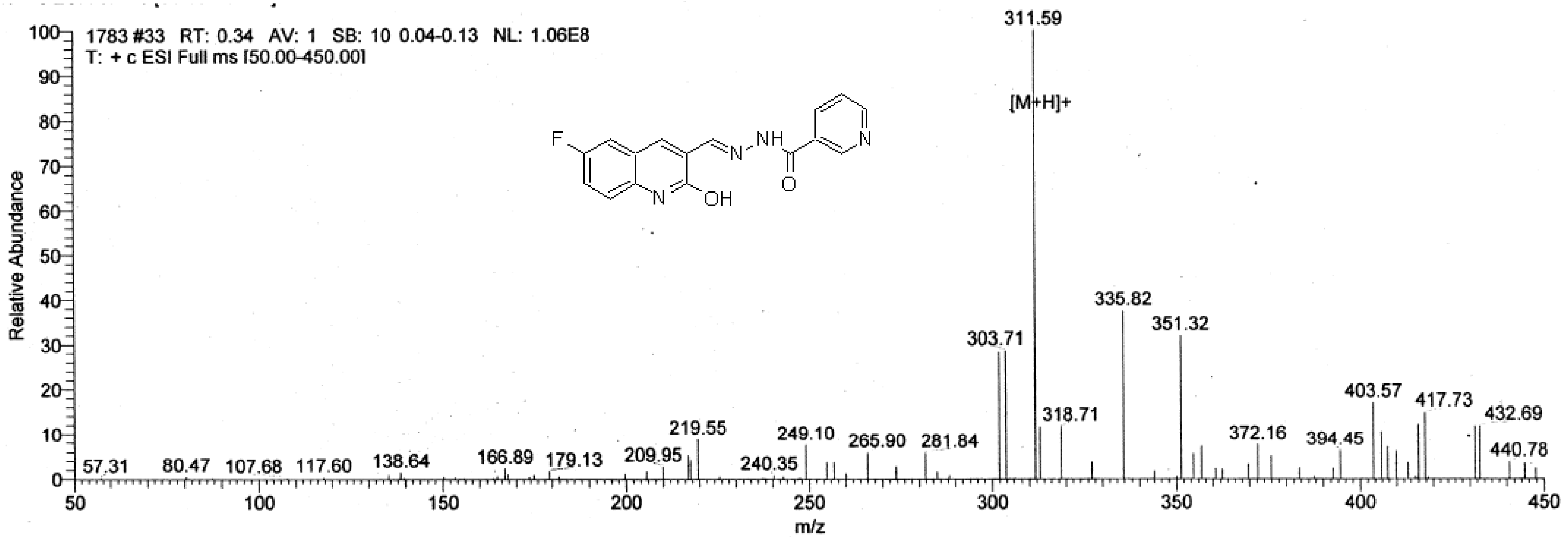

^{13}C NMR Spectra of [2a]

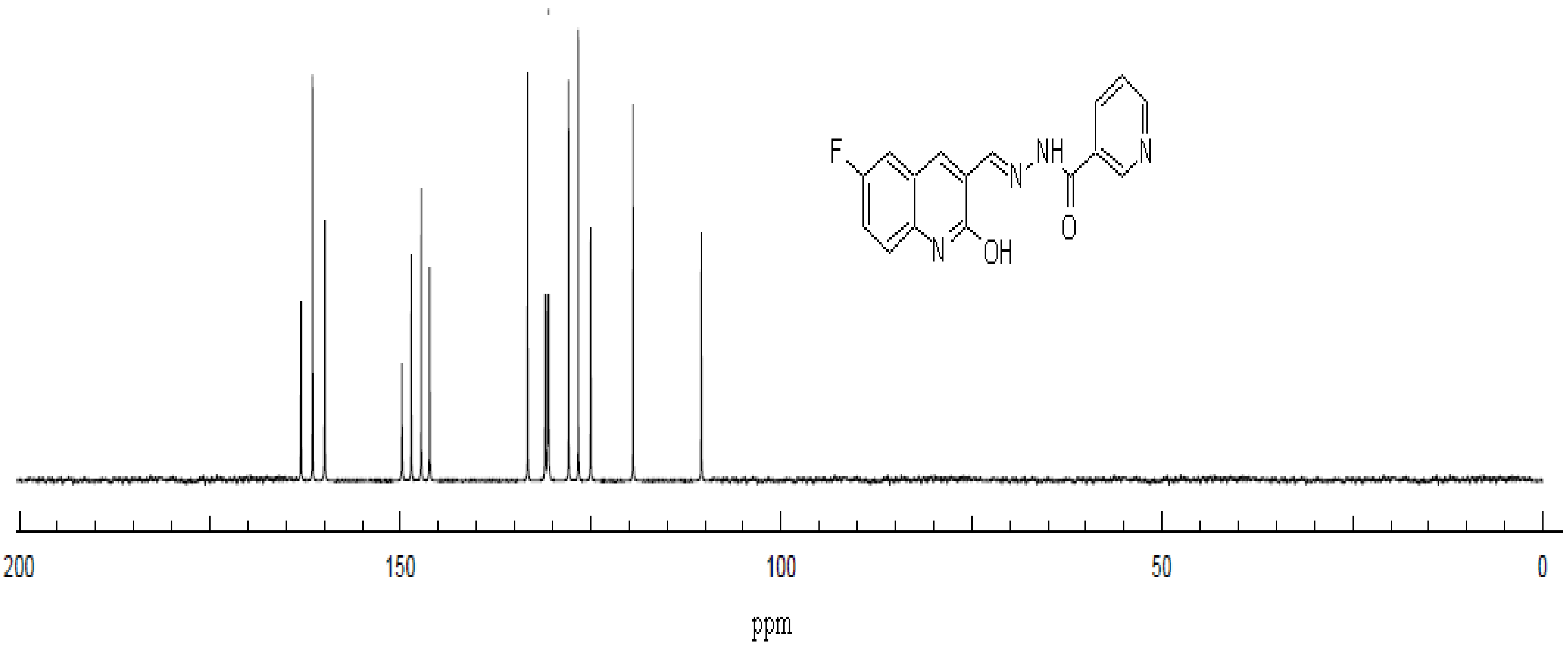

FTIR spectra of [2b]

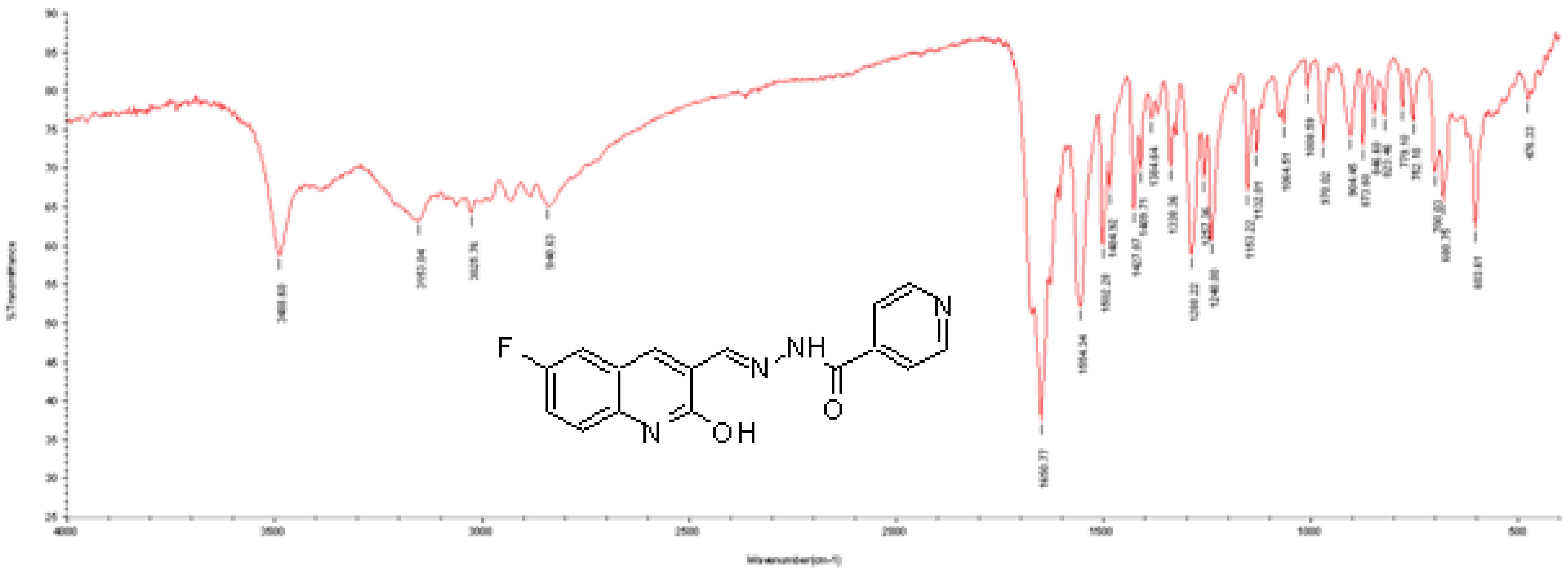

^{1}H NMR spectra of [2b]

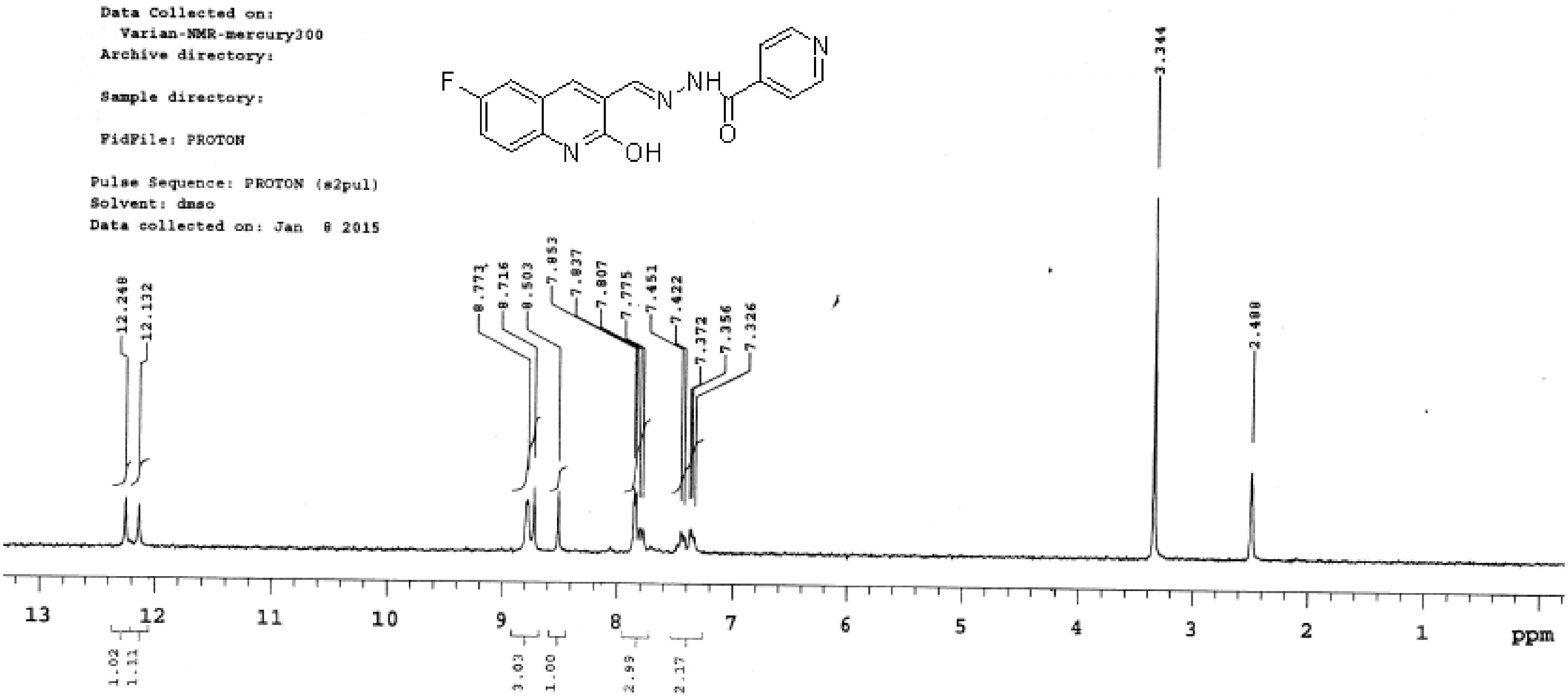

Mass spectra of [2b]

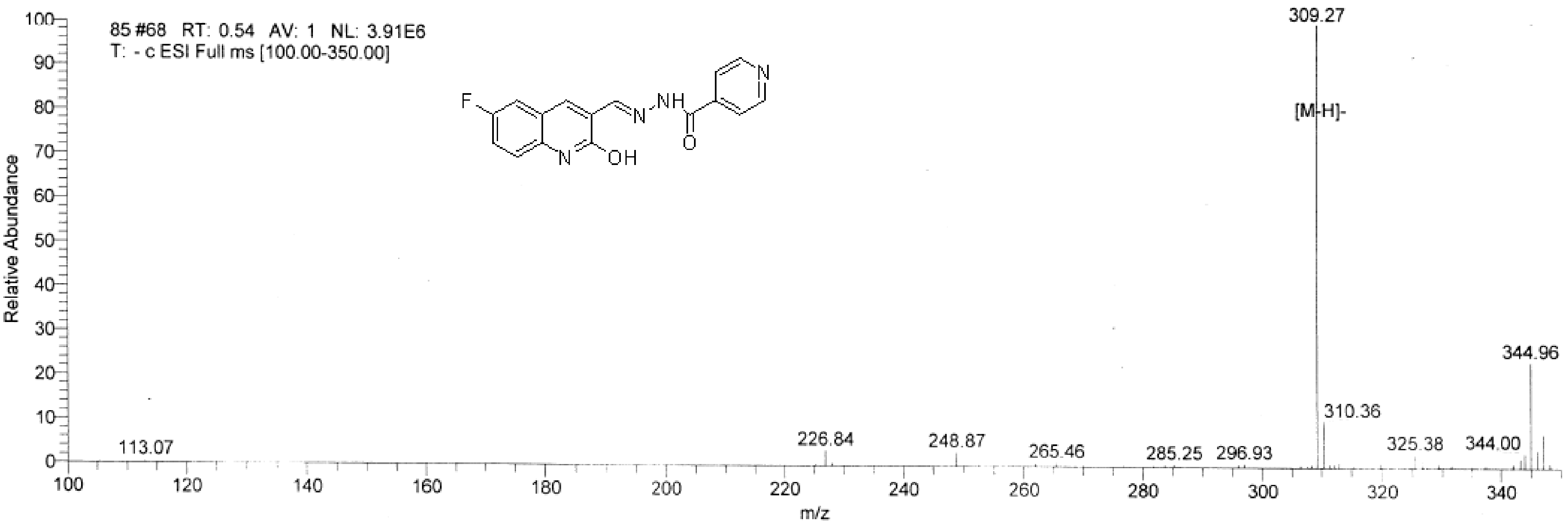

^{13}C NMR spectra of [2b]

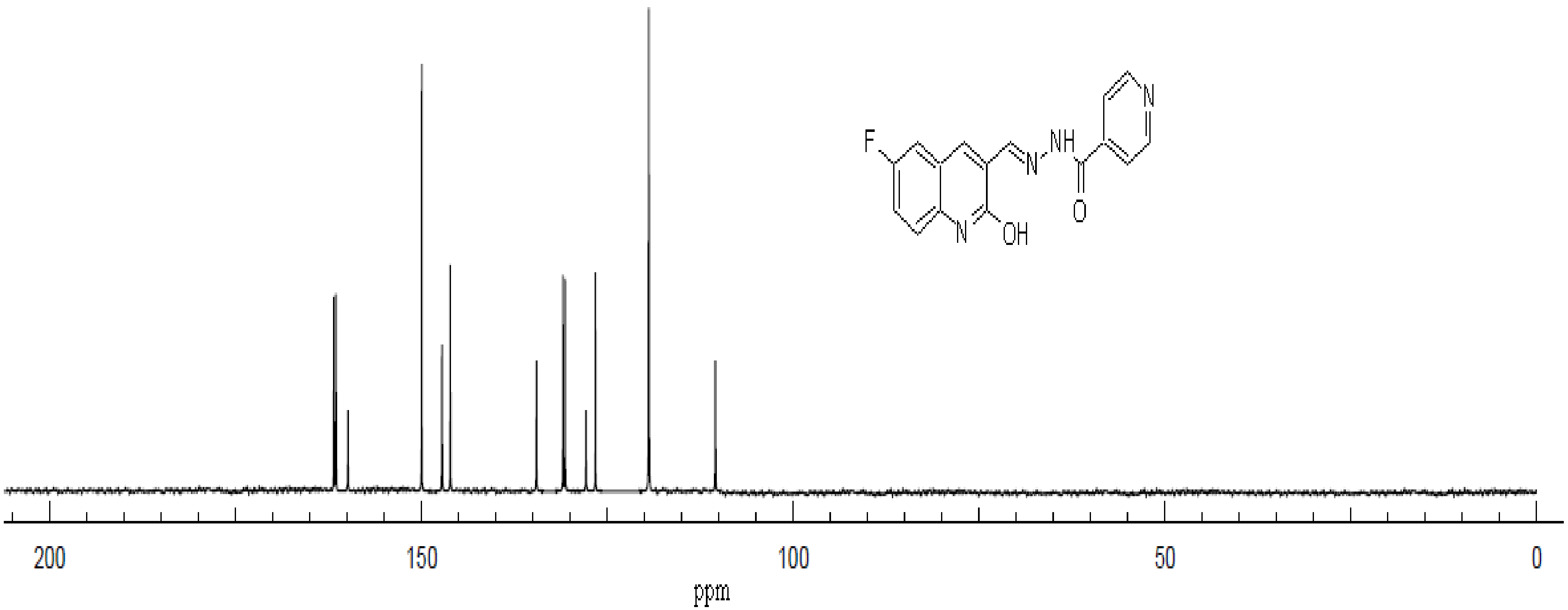

FTIR spectra of [2c]

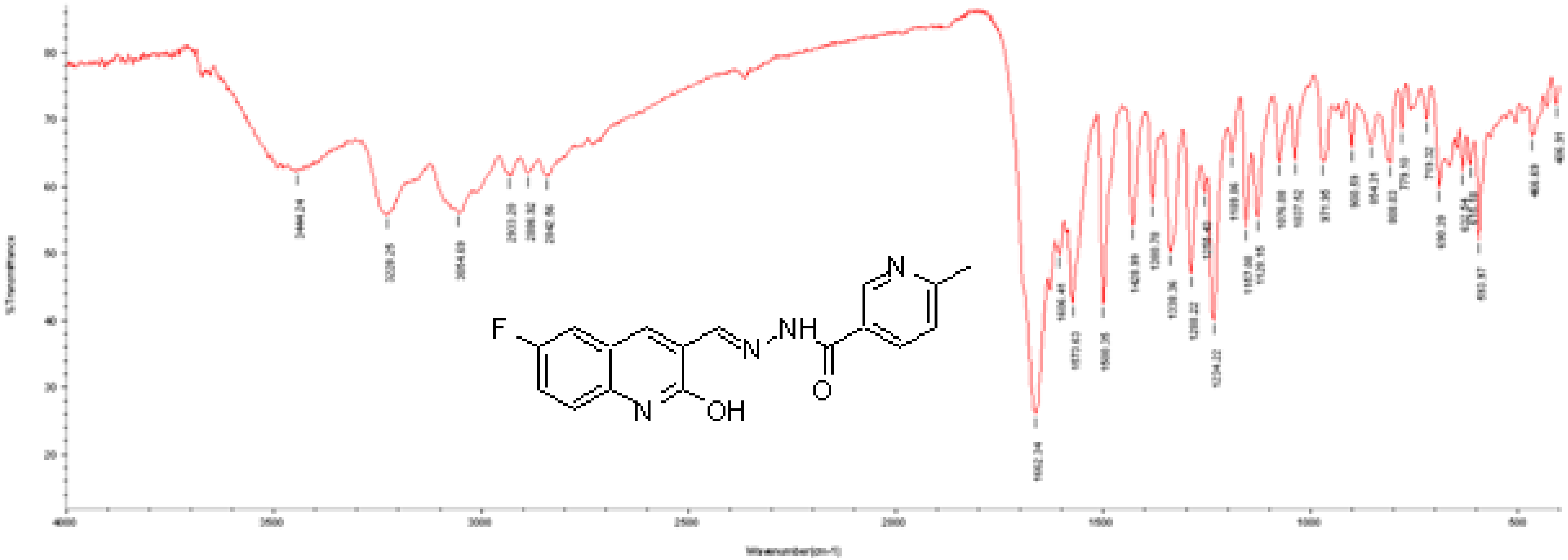

^{1}H NMR spectra of [2c]

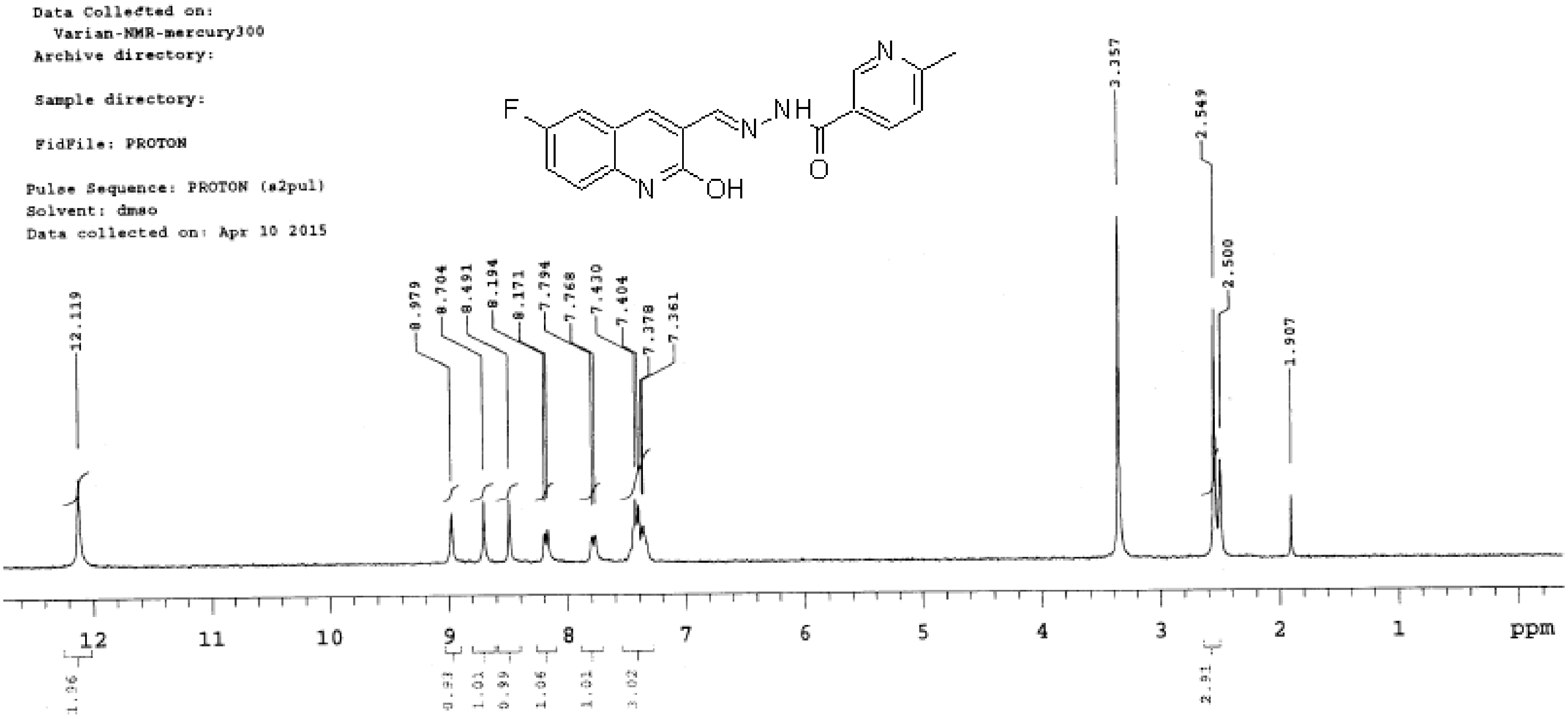

Mass spectra of [2c]

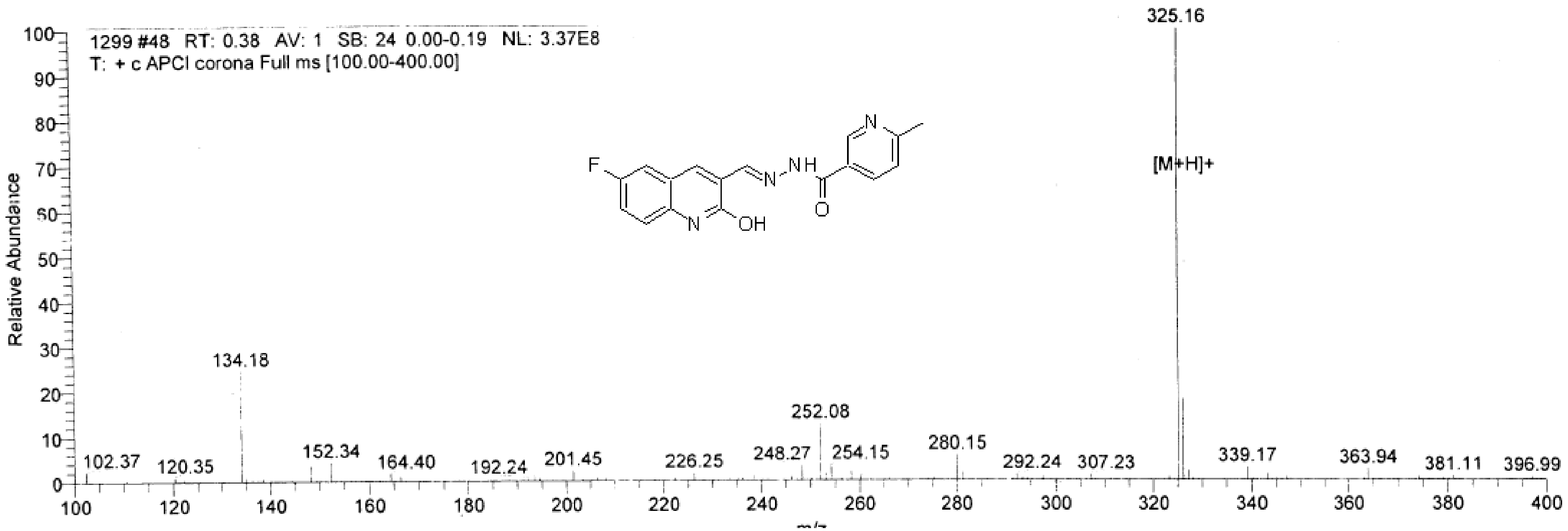

^{13}C NMR spectra of [2c]

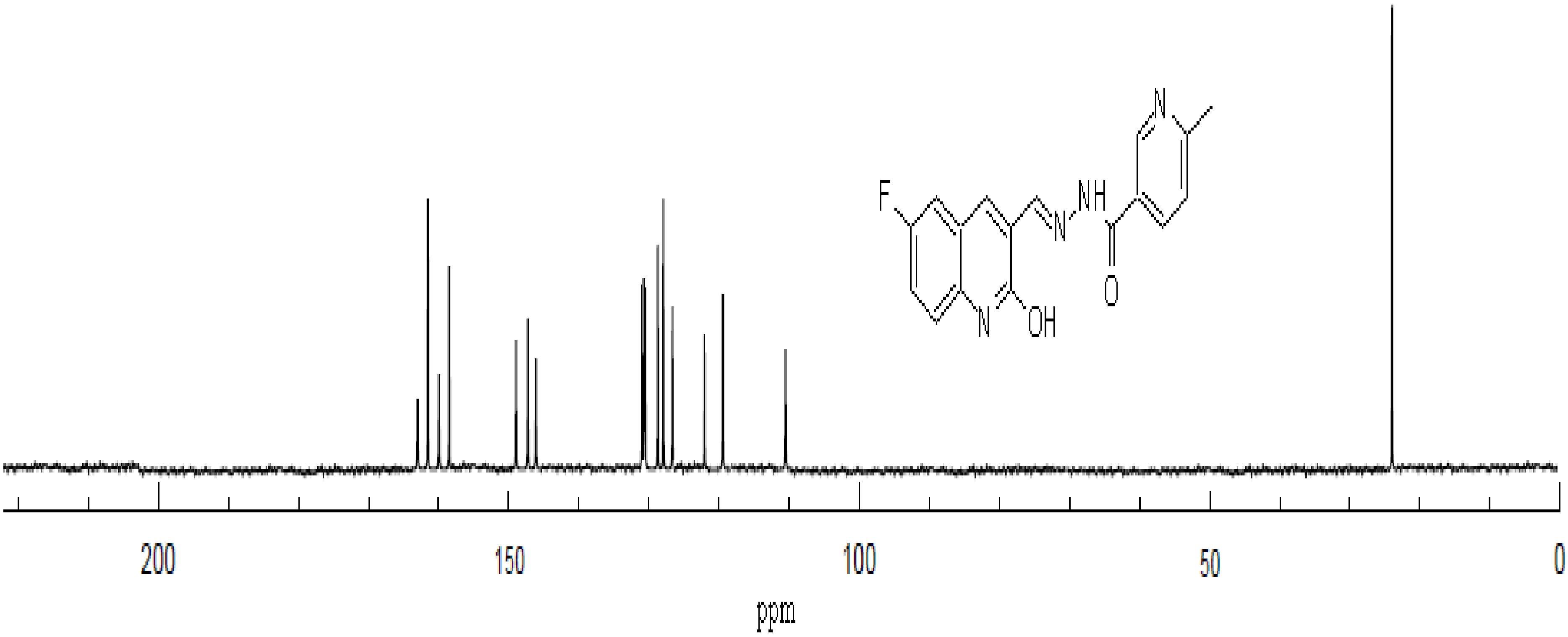

FTIR spectra of [2d]

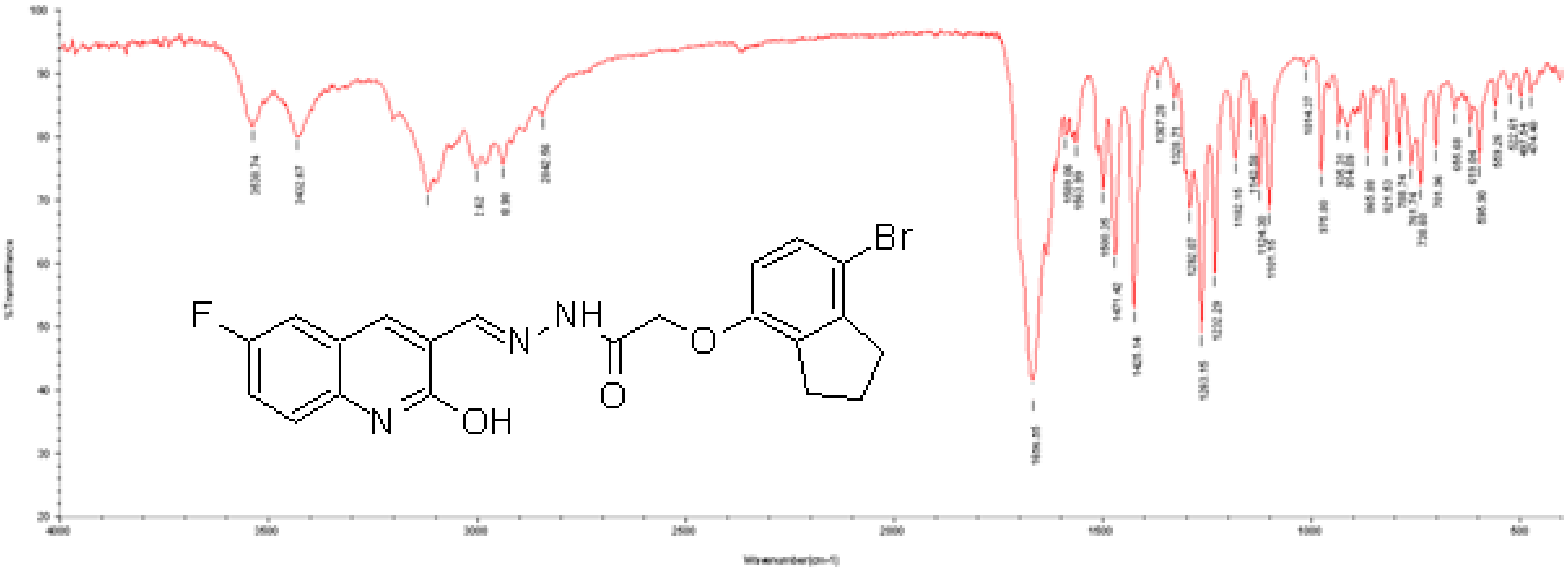

<u>**^{1}H NMR spectra of [2d]**</u>

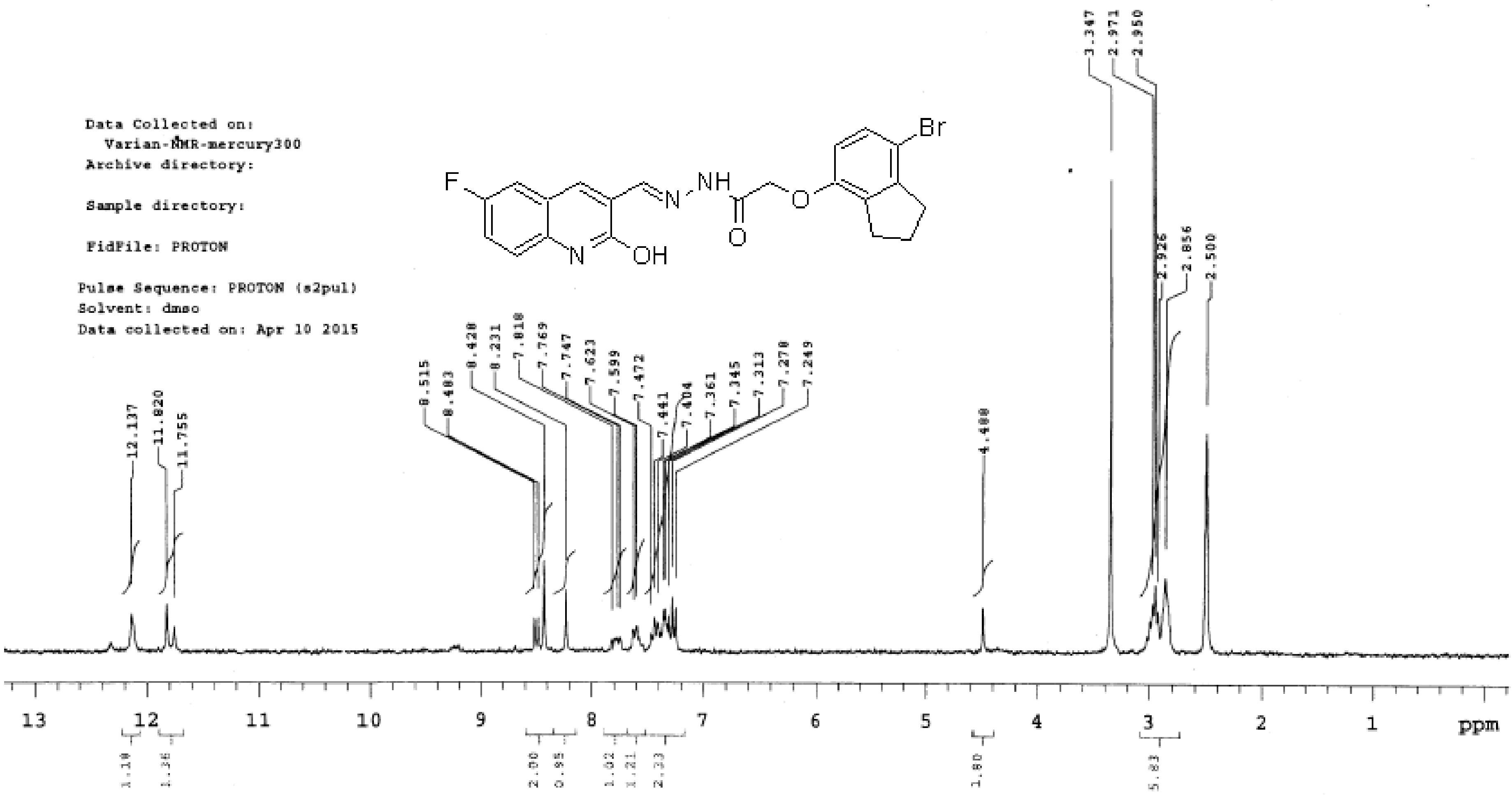

Mass spectra of [2d]

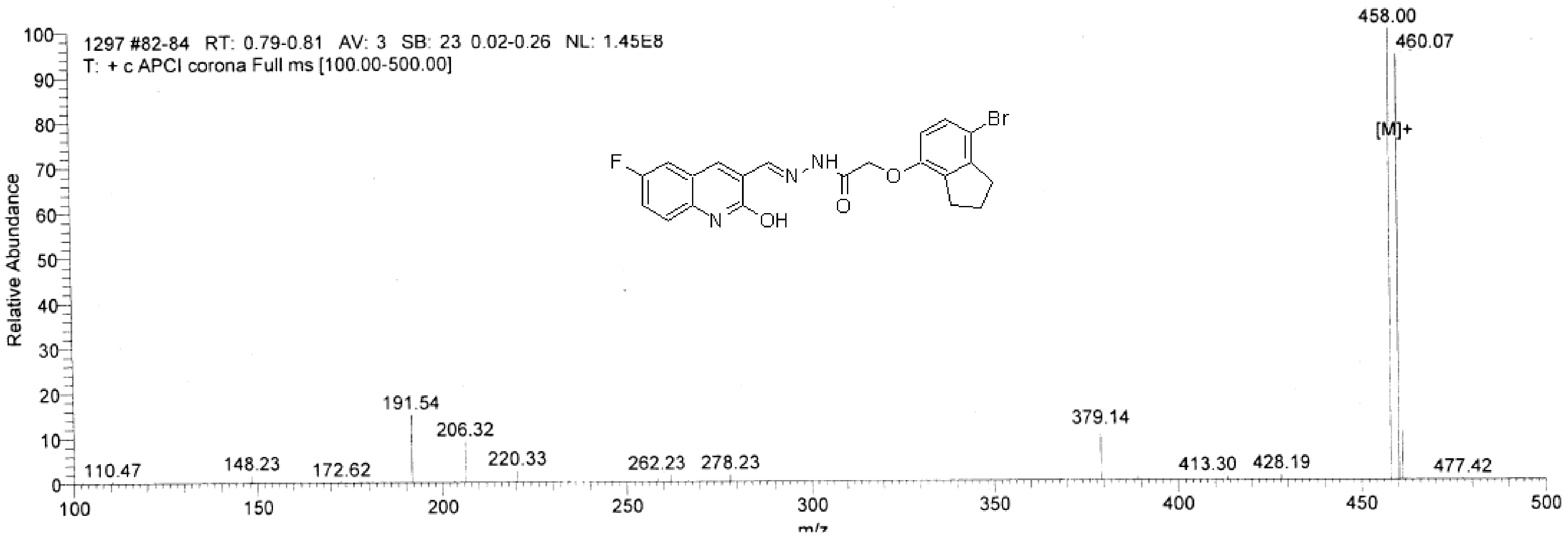

^{13}C NMR spectra of [2d]

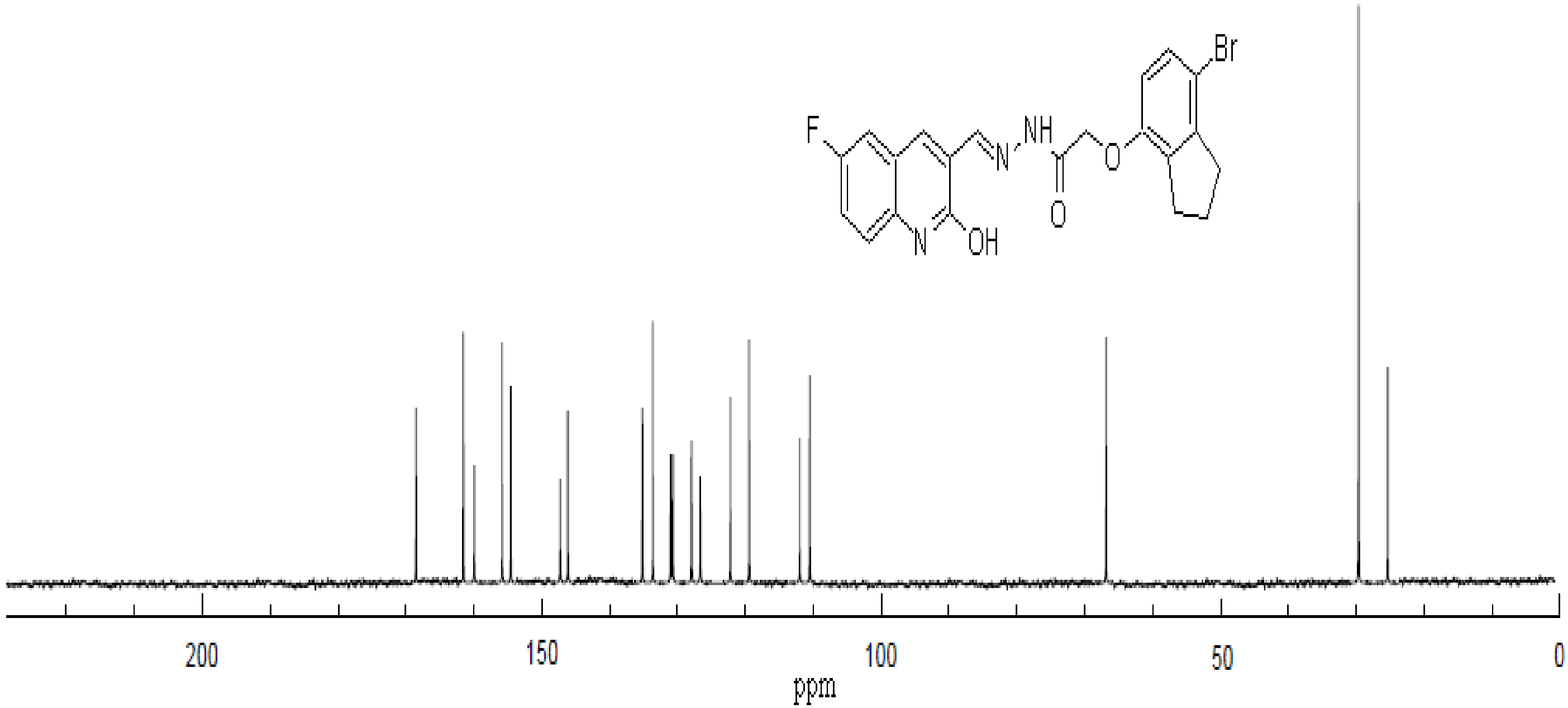

<u>**FTIR spectra of [2e]**</u>

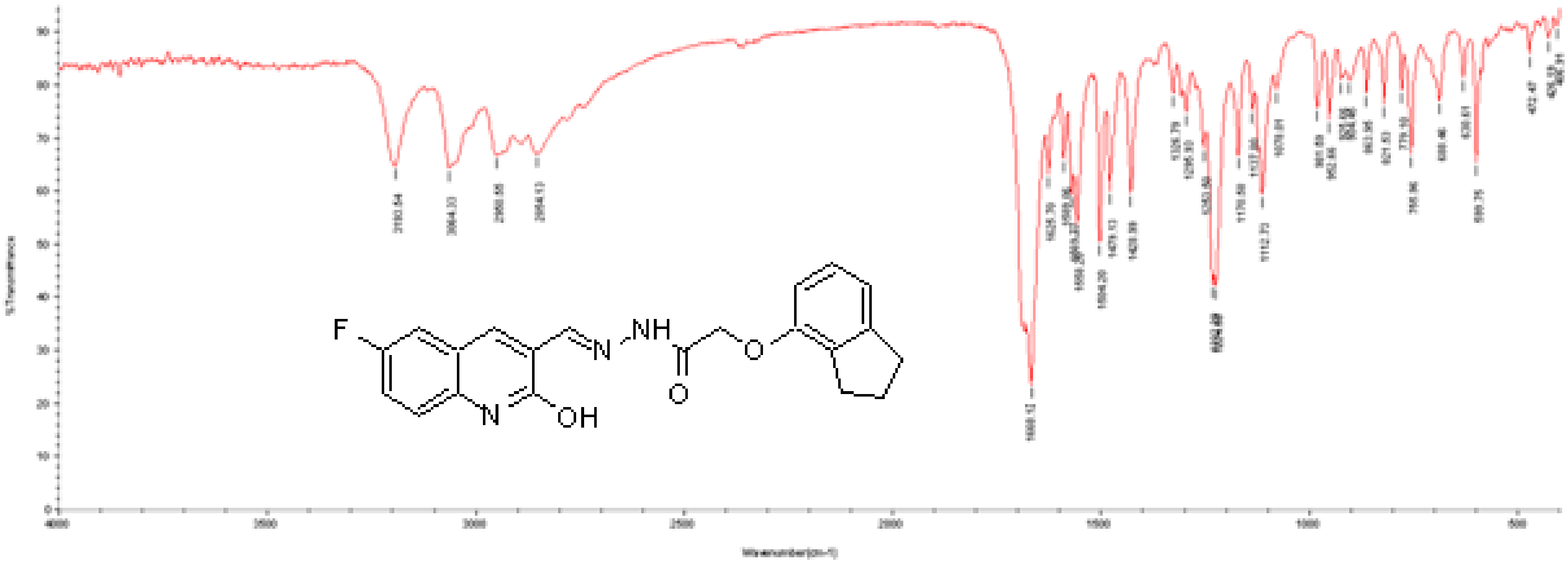

^{1}H NMR spectra of [2e]

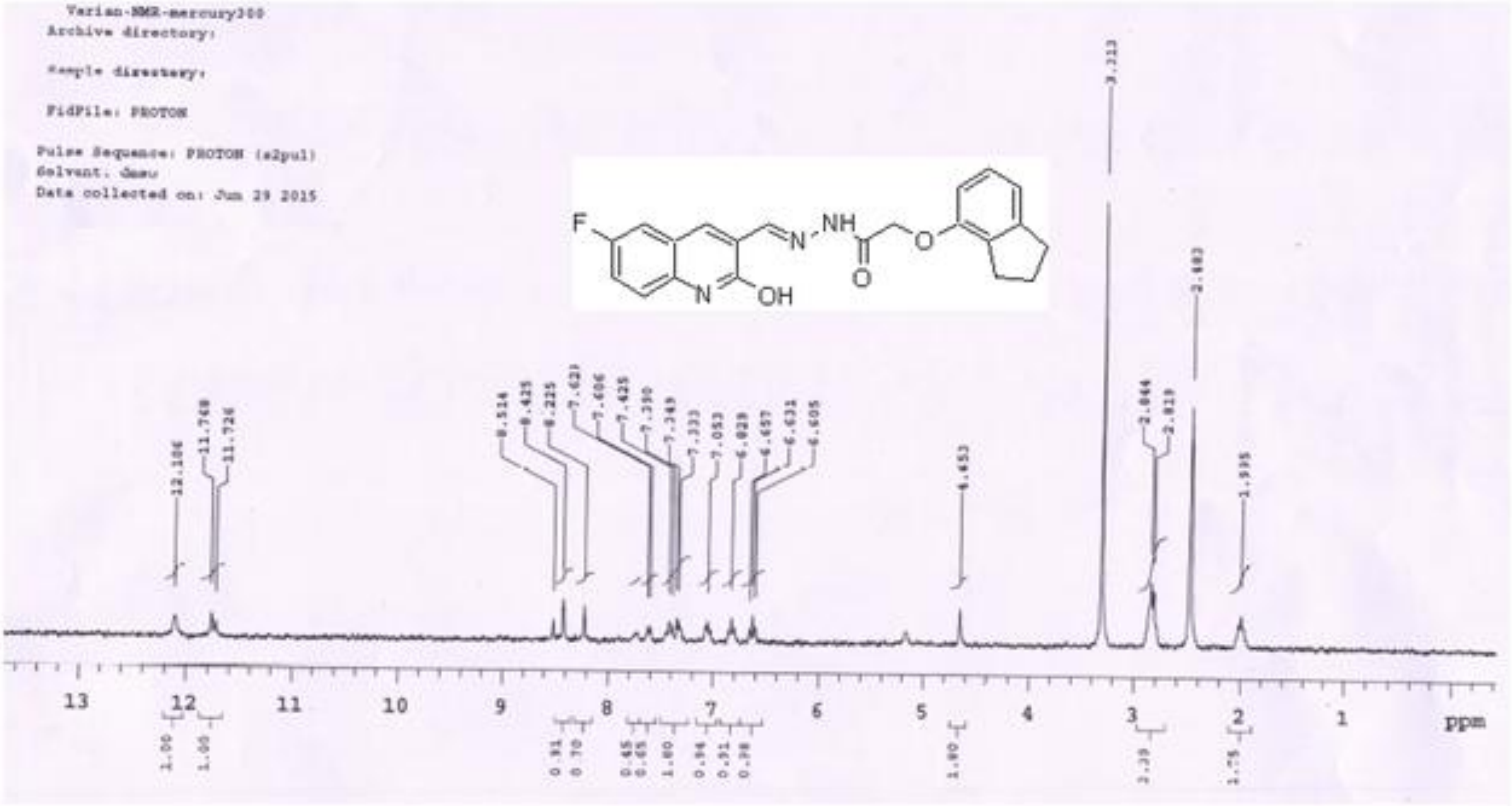

Mass spectra of [2e]

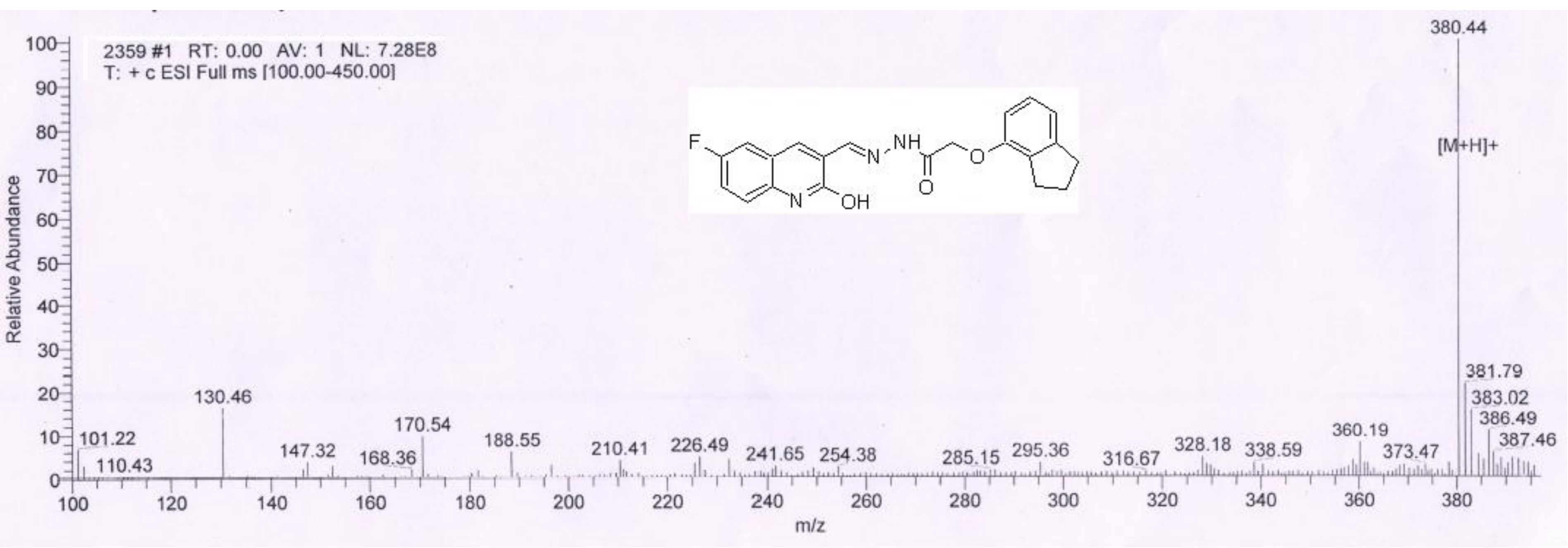

^{13}C NMR spectra of [2e]

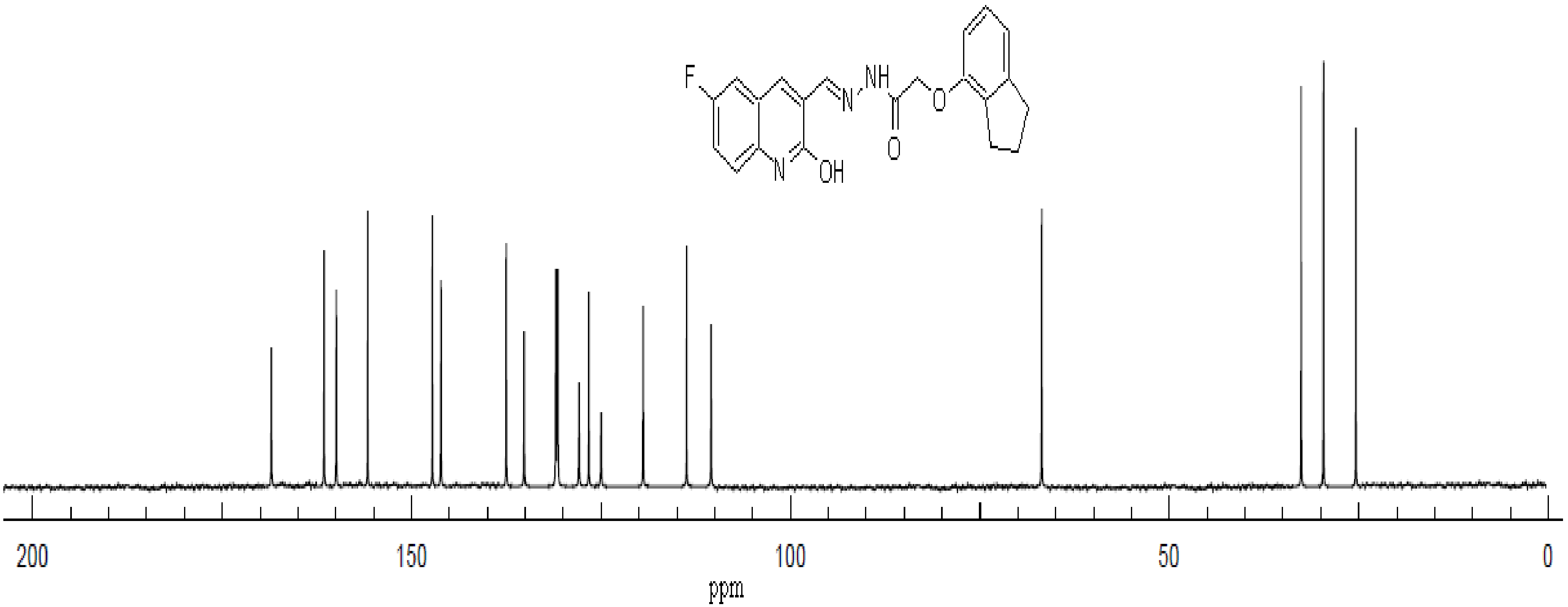

5. FTIR Spectra of metal complexes 3a-3j.

<u>FTIR Spectra of [3a]</u>

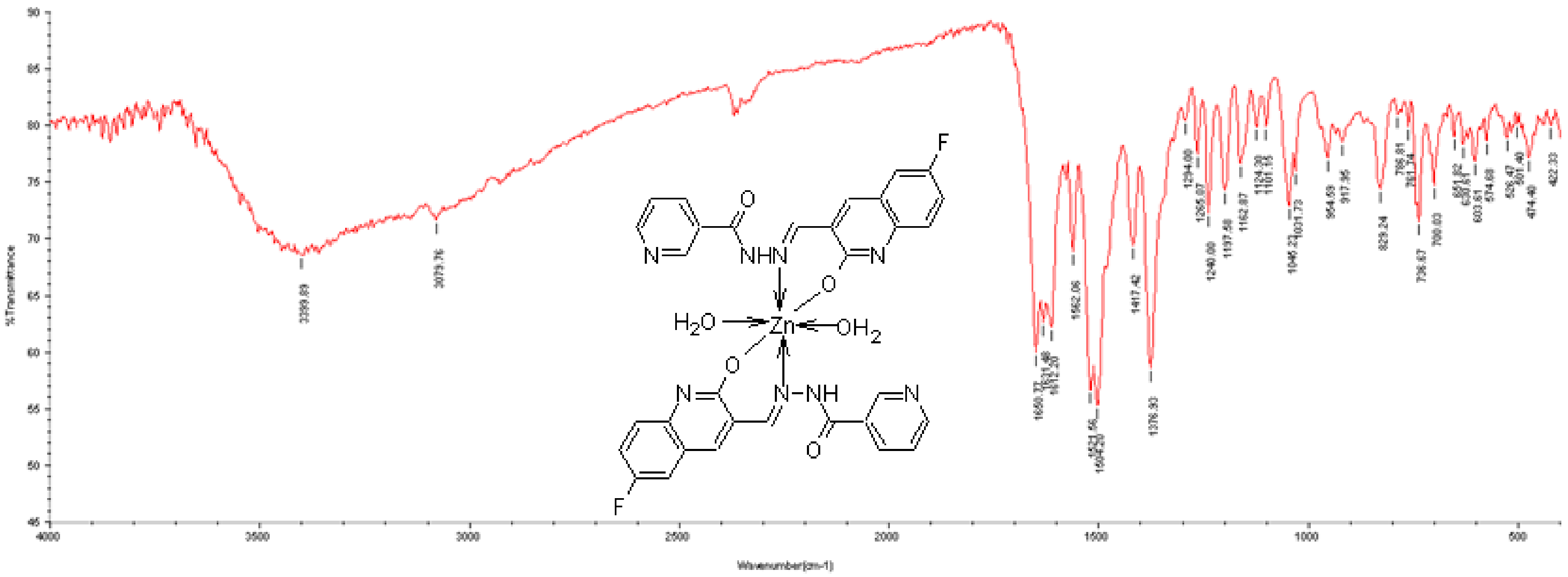

FTIR Spectra of [3b]

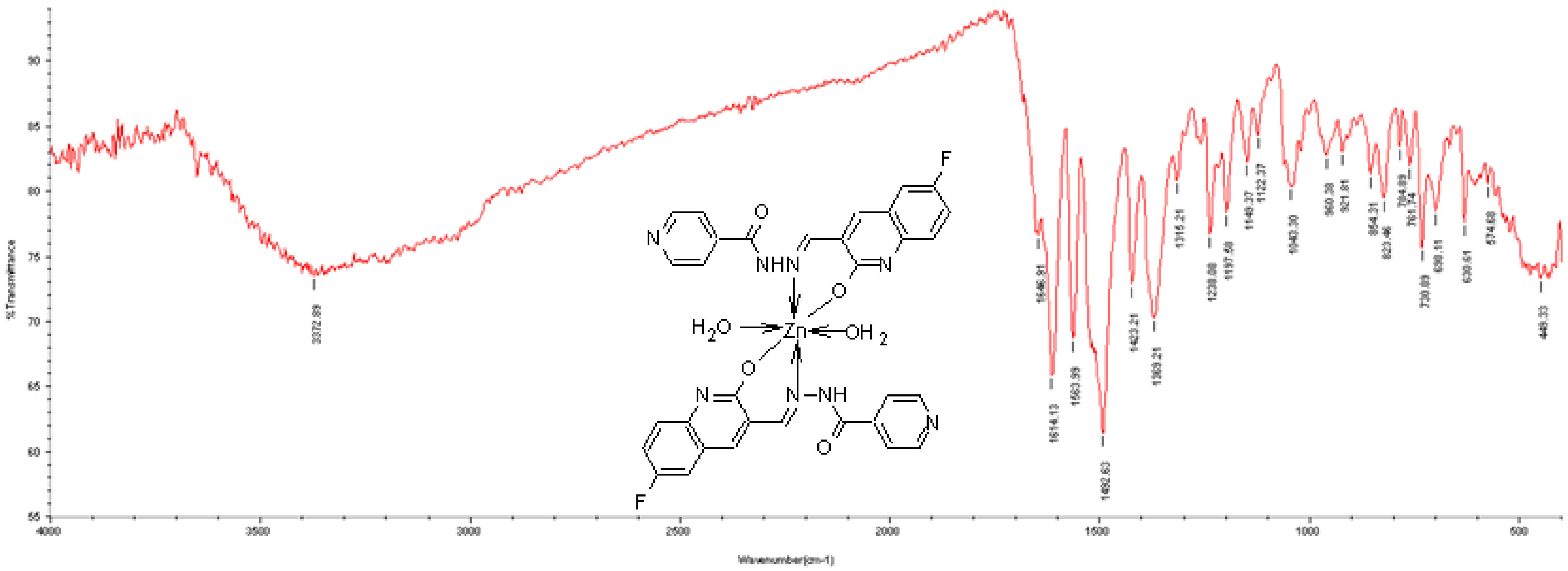

FTIR Spectra of [3c]

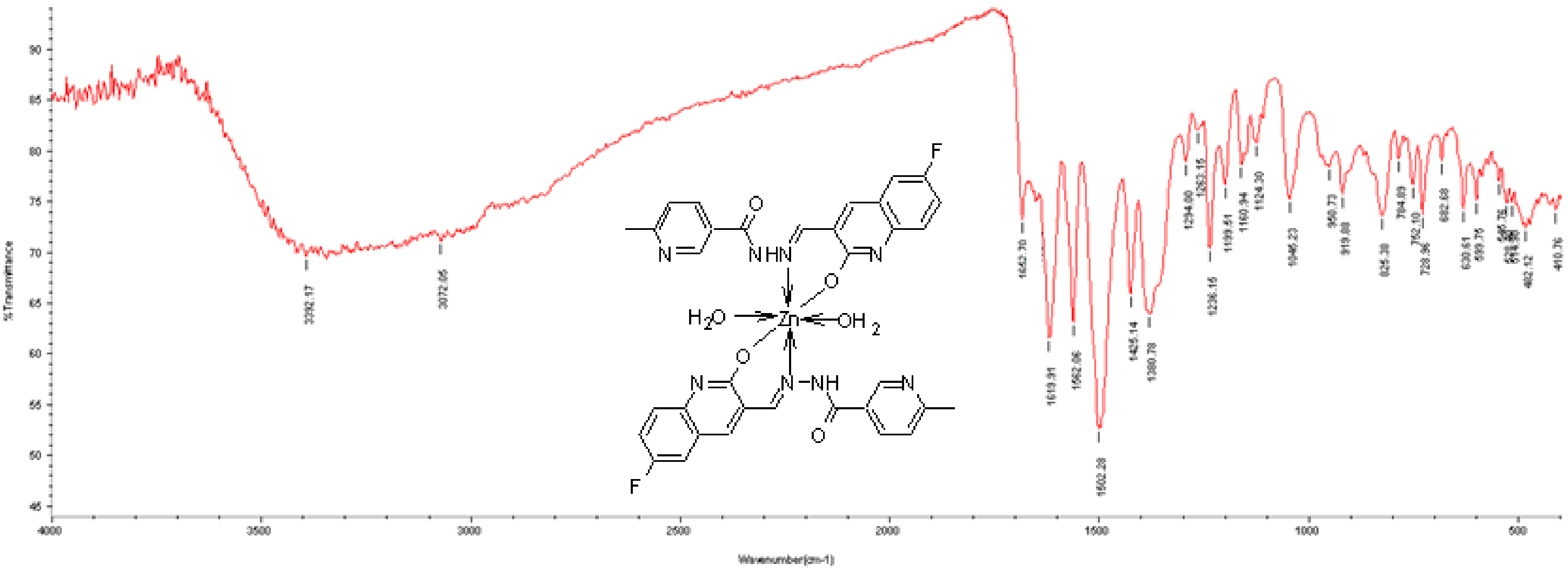

FTIR Spectra of [3d]

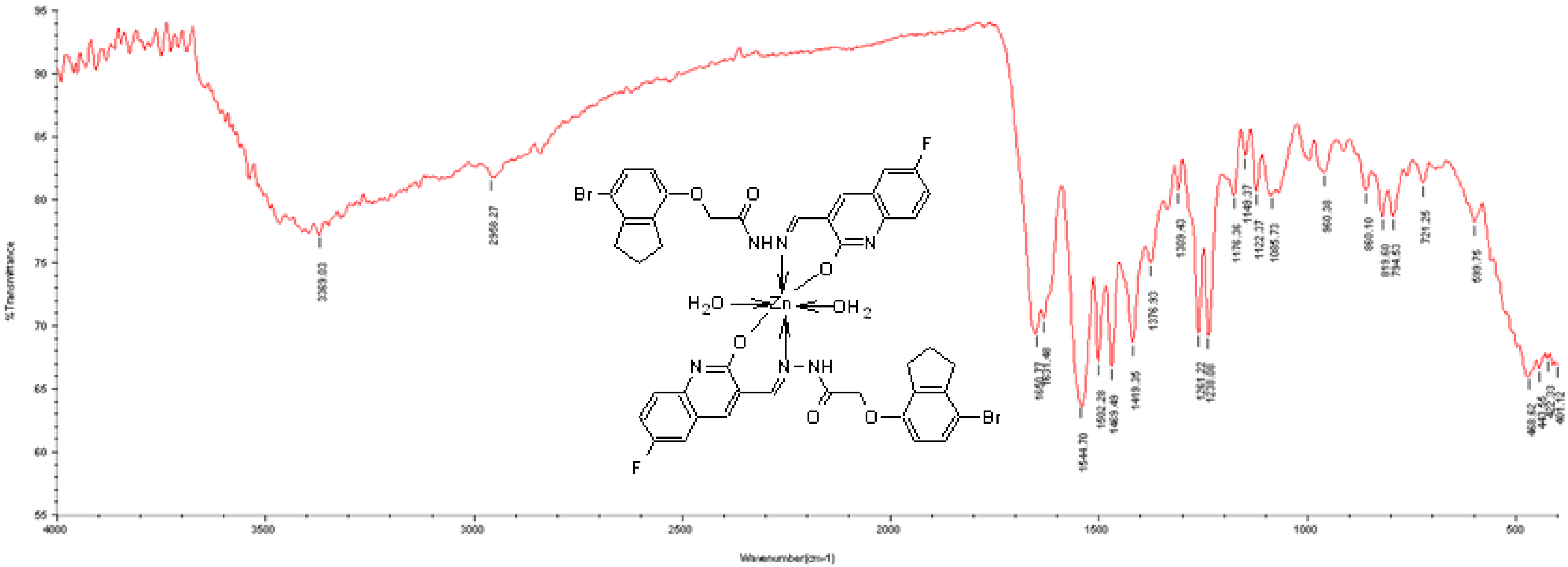

FTIR Spectra of [3e]

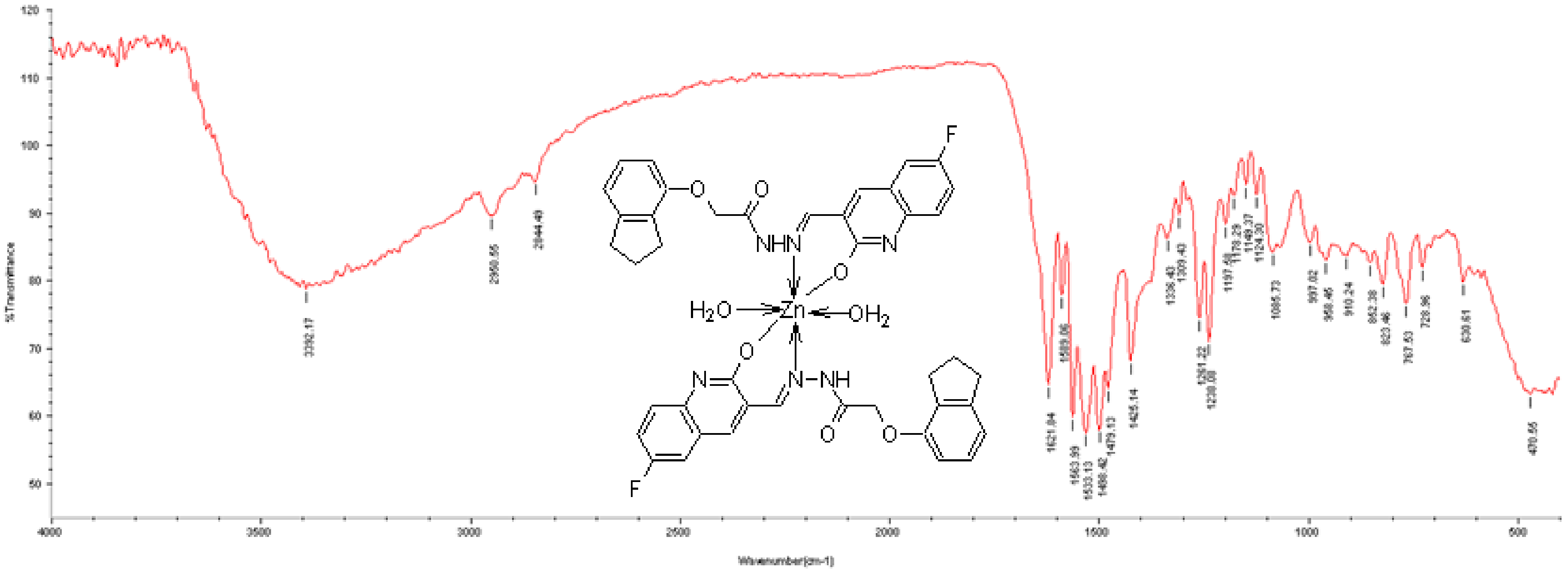

FTIR Spectra of [3f]

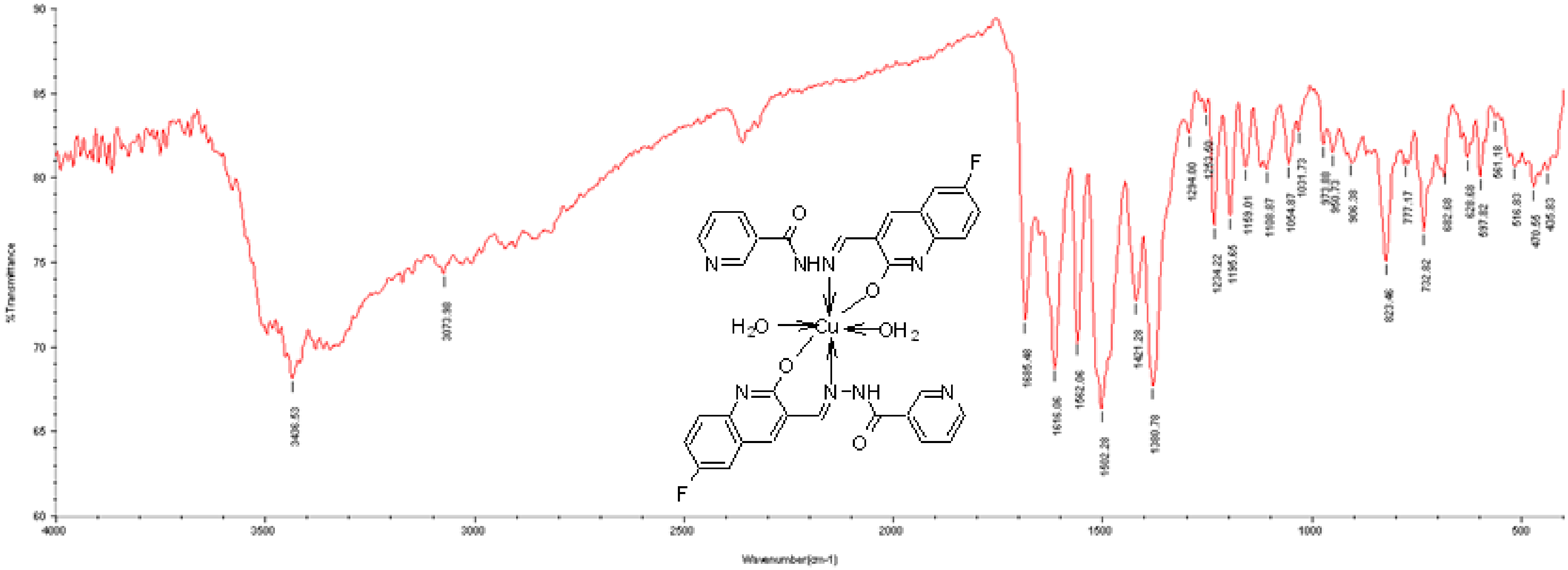

FTIR Spectra of [3g]

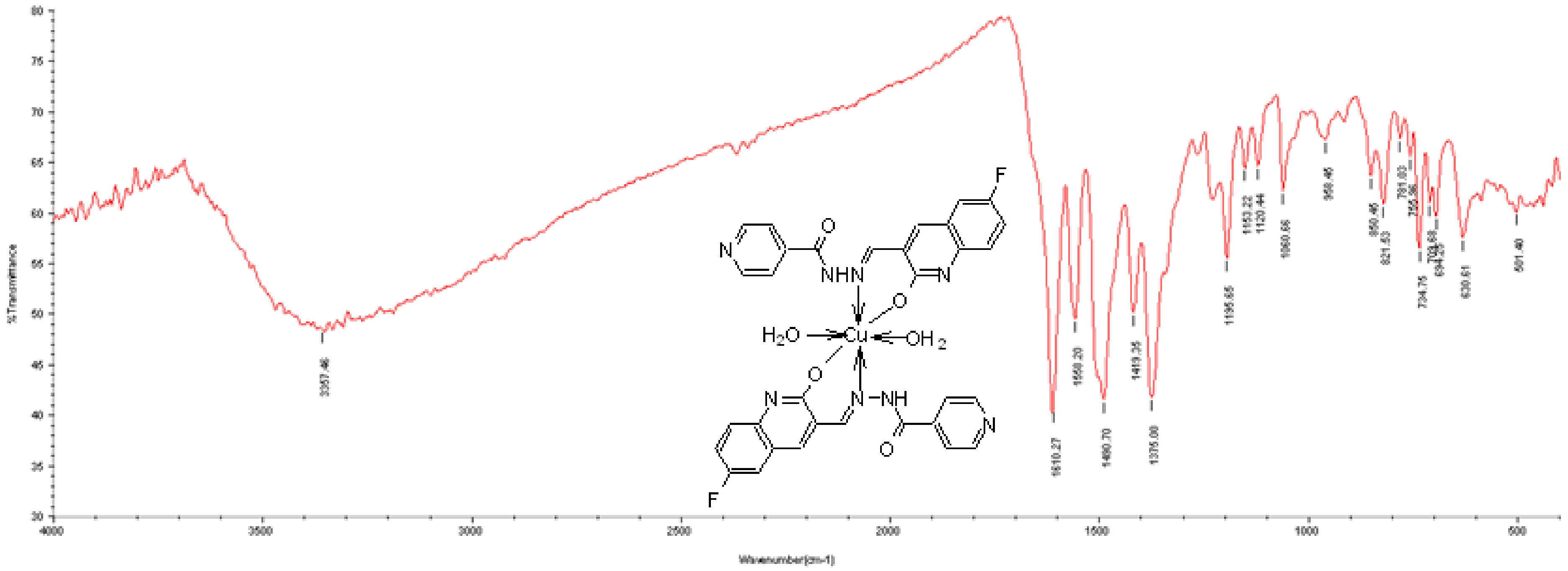

FTIR Spectra of [3h]

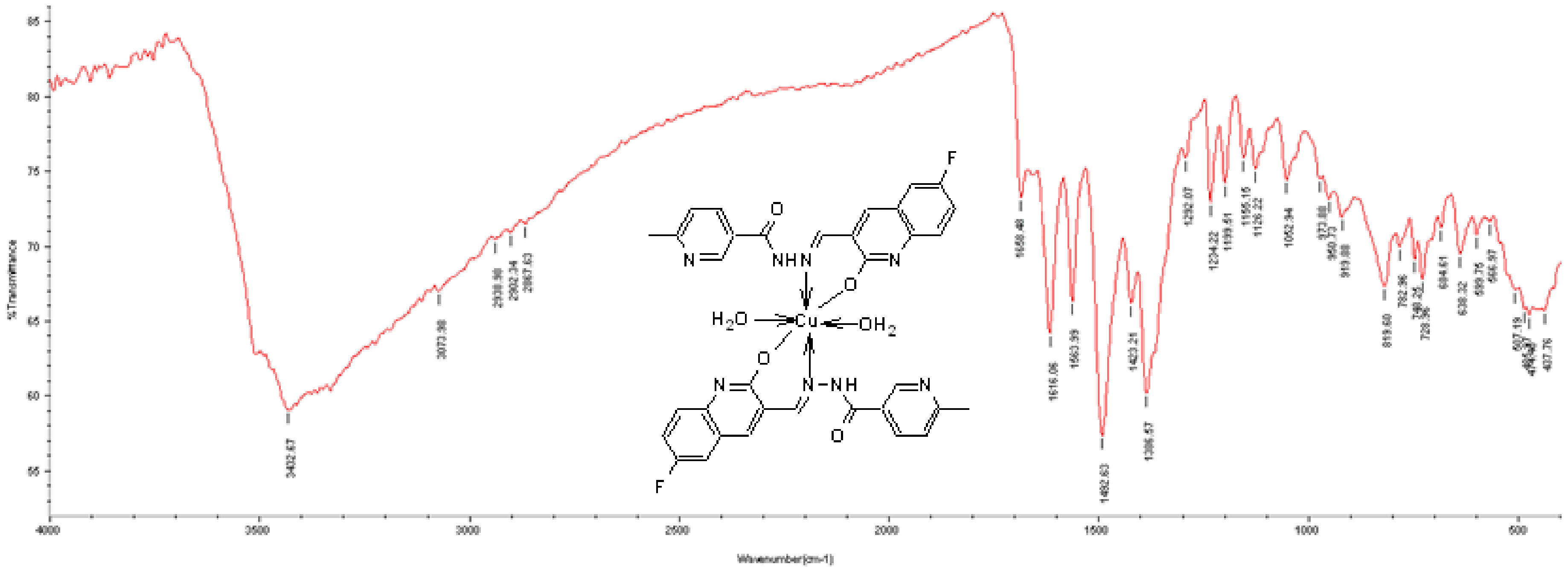

FTIR Spectra of [3i]

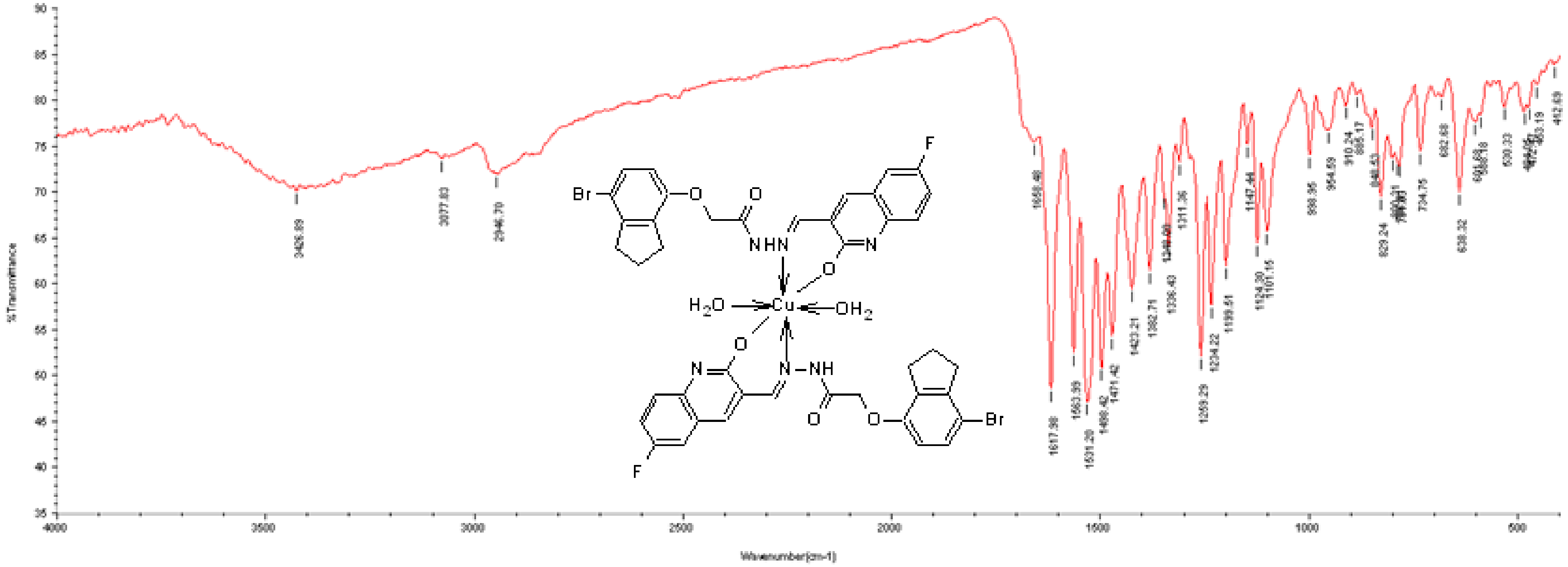

FTIR Spectra of [3j]

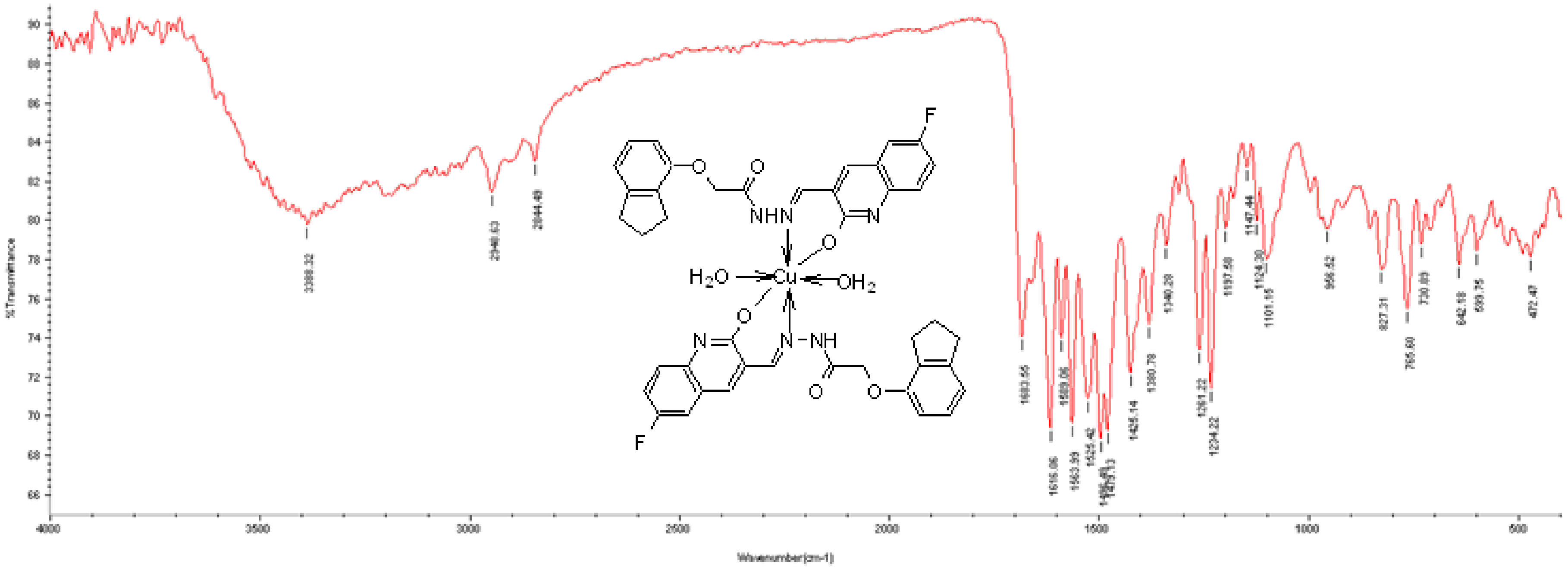

Supplement: Supplementary file 1 — D The material and methods utilised for the synthesis of hydrazone ligands and metal complexes provided in supplementary material. Structures of synthesized compounds and scanned images of spectras (1H, 13C NMR, FTIR, MASS) have been provided in supplementary material. [file 153015.f1.pdf]
